# A human tRNA methyltransferase 9-like protein prevents tumour growth by regulating LIN9 and HIF1-α

**DOI:** 10.1002/emmm.201201161

**Published:** 2013-02-04

**Authors:** Ulrike Begley, Maria Soledad Sosa, Alvaro Avivar-Valderas, Ashish Patil, Lauren Endres, Yeriel Estrada, Clement TY Chan, Dan Su, Peter C Dedon, Julio A Aguirre-Ghiso, Thomas Begley

**Affiliations:** 1 Division of Hematology and Oncology, Department of Medicine, Department of Otolaryngology, Tisch Cancer Institute, Mount Sinai School of MedicineNew York, NY; 2 Black Family Stem Cell Institute, Mount Sinai School of MedicineNew York, NY; 3 College of Nanoscale Science and Engineering, University at Albany, State University of New YorkAlbany, NY; 4 Department of Biomedical Sciences, School of Public Health, University at Albany, State University of New YorkAlbany, NY; 5 Department of Chemistry, Massachusetts Institute of TechnologyCambridge, MA; 6 Department of Biological Engineering, Massachusetts Institute of TechnologyCambridge, MA; 7 Center for Environmental Health Sciences, Massachusetts Institute of TechnologyCambridge, MA

**Keywords:** cancer, hTRM9L, hypoxia, translation, tRNA modification

## Abstract

Emerging evidence points to aberrant regulation of translation as a driver of cell transformation in cancer. Given the direct control of translation by tRNA modifications, tRNA modifying enzymes may function as regulators of cancer progression. Here, we show that a tRNA methyltransferase 9-like (*hTRM9L/KIAA1456*) mRNA is down-regulated in breast, bladder, colorectal, cervix and testicular carcinomas. In the aggressive SW620 and HCT116 colon carcinoma cell lines, *hTRM9L* is silenced and its re-expression and methyltransferase activity dramatically suppressed tumour growth *in vivo*. This growth inhibition was linked to decreased proliferation, senescence-like G0/G1-arrest and up-regulation of the RB interacting protein LIN9. Additionally, SW620 cells re-expressing *hTRM9L* did not respond to hypoxia via HIF1-α-dependent induction of GLUT1. Importantly, *hTRM9L*-negative tumours were highly sensitive to aminoglycoside antibiotics and this was associated with altered tRNA modification levels compared to antibiotic resistant *hTRM9L*-expressing SW620 cells. Our study links hTRM9L and tRNA modifications to inhibition of tumour growth via LIN9 and HIF1-α-dependent mechanisms. It also suggests that aminoglycoside antibiotics may be useful to treat hTRM9L-deficient tumours.

## INTRODUCTION

The regulation of translation has primarily been studied at the level of initiation, with both CAP-dependent and CAP-independent regulatory mechanisms playing vital roles in cellular proliferation, stress signalling and cell cycle progression (Komar & Hatzoglou, 2011; Sonenberg & Hinnebusch, 2009; Sonenberg et al, 2000). Translation elongation is another regulatory strategy and, at its core, requires the efficient interaction of codons and anticodons found on mRNA and tRNA, respectively. Enzyme catalysed modification of tRNA has been shown to dramatically affect codon–anticodon interactions (Murphy et al, 2004; Yarian et al, 2002). Among the tRNA modifications in eukaryotes, wobble uridine modifications have been well studied in *Saccharomyces cerevisiae*, with 11 of 13 tRNAs possessing either 5-carbamoylmethyluridine (ncm^5^U), the 2′-O-ribose-methylated form ncm^5^Um, 5-methoxycarbonylmethyluridine (mcm^5^U) or 5-methoxycarbonylmethyl-2-thiouridine (mcm^5^s^2^U; Johansson et al, 2008). Wobble uridine and anticodon-associated modifications on tRNA molecules promote or restrict specific anticodon–codon interactions and can influence translation speed and fidelity. In fact, the levels of many modifications associated with the structure of the anticodon (positions 34 or 37) change in response to cellular stress, supporting that translation elongation is dynamically regulated (Chan et al, 2010).

**Figure 1:**
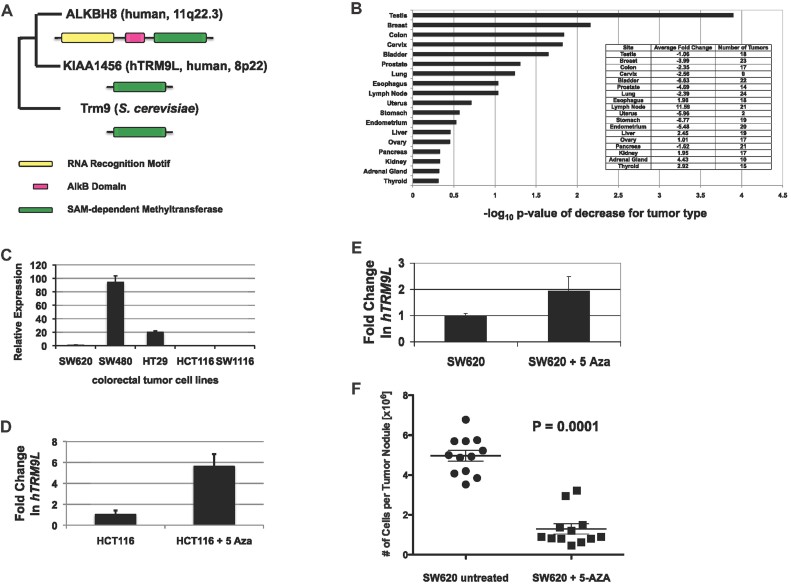
*hTRM9L* transcripts are decreased in many tumour types and the tumourgenicity of SW620 colon cancer cells can be decreased by treatment with a 5-meC demethylating agent **A.** Annotated gene tree of yeast Trm9 homologs. Two yTrm9 homologs are found in humans, *KIAA1456*(*hTRM9L*) and *hALKBH8*. In addition to the SAM-dependent Methyltransferase domain, hALKBH8 contains a RNA Recognition Motif and an AlkB domain.**B.** *KIAA1456* (*hTRM9L*) expression profiling of Origene Tissue Scan qPCR array covering 18 different cancer types was used to identify significant differences in expression of *hTRM9L* in these cancer tissues compared to normal tissues. Significant differences in *hTRM9L* transcripts were evaluated and scored (Log10 *p*-value).**C.** *hTRM9L* expression was analysed in various colorectal cancer cell lines. RNA was isolated from SW620, SW480, HT29, HCT116 and SW1116 cell lines and *hTRM9L* transcript levels were quantitated (ΔΔ*C*_T_ method) by qPCR.**D,E.** HCT116 or SW620 cells were treated with 10 µM 5-aza-dC over a period of 7 days with the culture being replaced every 24 h with fresh containing 5-aza-dC. RNA was isolated and *hTRM9L* expression was determined by qPCR analysis.**F.** Mock or 5-aza-dC treated SW620 cells (5 × 10^5^) were inoculated on CAM and grown for 7 days *in vivo*. Tumours were excised, minced and collagenased and the number of cells per tumour nodule was counted. Statistical significance determined by paired student's *t*-test.

The completion of the wobble uridine modification mcm^5^U (a precursor for mcm^5^s^2^U) is catalysed by yeast tRNA methyltransferase 9 (Trm9) and its mammalian homolog ALKBH8 (Begley et al, 2007; Fu et al, 2010a; Songe-Moller et al, 2010). Amino acid sequence analyses reveal one gene encoding Trm9 in *S. cerevisiae* (Trm9) and two Trm9 homologs in mammals (KIAA1456 and ALKBH8; Fig 1A). Trm9 has been biochemically characterized. After elongator complex proteins generate the substrate cm^5^U, Trm9 uses the methyl donor *S*-adenosyl methionine (SAM) to complete the formation of mcm^5^-based modifications. Trm9 forms a complex with Trm112 to catalyse the last step in the formation of mcm^5^U (Mazauric et al, 2010). Clarke and coworkers initially characterized Trm9 and demonstrated that it modifies wobble uridines in specific arginine and glutamic acid tRNAs. They further showed that Trm9-deficient yeast cells were sensitive to the translational infidelity inducing aminoglycoside antibiotic paromomycin (Kalhor & Clarke, 2003). Cells deficient in Trm9 also display sensitivity to DNA damaging agents (*e.g.* MMS and IR) and have a damage induced cell cycle progression defect (Begley et al, 2002, 2004, 2007; Bennett et al, 2001; Chan et al, 2010).

ALKBH8 is the most thoroughly characterized mammalian homolog of yeast Trm9 and ALKBH8 deficient cells are sensitive to DNA damaging agents (Fu et al, 2010a). ALKBH8 makes the wobble uridine modifications mcm^5^U and mchm^5^U. The formation of mcm^5^U is required for the completion of the mcm^5^s^2^U, and mcm^5^Um modifications (Fu et al, 2010a, b; Songe-Moller et al, 2010; van den Born et al, 2011). Mouse ALKBH8 has also been implicated in the recoding of stop codons to promote the incorporation of selenocysteine into specific proteins (Songe-Moller et al, 2010). Compared to yeast Trm9, ALKBH8 contains additional 2-oxoglutarate- and iron-dependent dioxygenase and RNA binding domains. The second yeast Trm9 homolog identified in mice and humans is KIAA1456, but there is little functional information associated with the corresponding proteins. We have tentatively designated KIAA1456 as hTRM9L (human TRM9-like protein). The *hTRM9L* gene encodes a protein that contains an SAM-dependent methyltransferase domain. Based on domain structure and protein size hTRM9L is similar to yeast Trm9. In humans, the *hTRM9L* gene maps to the end of human chromosome 8, a region commonly lost or silenced in many different cancers, including colorectal carcinoma (Ilyas et al, 1999; Kerangueven et al, 1995; Knowles et al, 1993; Prasad et al, 1998). Recent studies have implicated *hTRM9L* as a potential tumour suppressor gene (Flanagan et al, 2004). These studies, conducted in soft agar, demonstrated that a 250 mBp piece of DNA specific to the end of chromosome 8, where *hTRM9L* and other genes are located, decreased the colony formation of specific colorectal cancer lines.

Wobble base modifications catalysed by yeast Trm9 and ALKBH8 proteins play important roles in stress signalling pathways, with responses to DNA damage and reactive oxygen species as prime examples (Begley et al, 2007; Chan et al, 2010; Fu et al, 2010a; Songe-Moller et al, 2010). The potential presence of a tumour suppressor on chromosome 8, in a region that encodes *hTRM9L*, and the linkage between tumour suppressors and stress signalling pathways led us to postulate that hTRM9L could play a role in controlling tumour growth. In this study, we show that hTRM9L is a powerful negative regulator of tumour growth and that the *hTRM9L* transcript is significantly down-regulated in breast, bladder, cervix, testicular and colorectal carcinomas. Further, we demonstrate that the down-regulation of *hTRM9L* is due to epigenetic silencing in advanced colorectal cancer cell lines. Importantly, re-expression of *hTRM9L* strongly inhibits SW620 and HCT116 colon carcinoma cell tumourigenicity *in vivo* via a senescence-like G0/G1-arrest. Further, we show that inhibition of tumour growth by hTRM9L is dependent on a functional SAM binding domain. Tumour growth inhibition by hTRM9L is linked to increased transcription of the RB interacting protein LIN9 and to a failure of hTRM9L-expressing cells to mount a hypoxic response. We also demonstrate that the hTRM9L expressing cells have a significant increase in mcm^5^U and other tRNA modifications after paromomycin treatment, relative to SW620-LacZ and that hTRM9L promotes global changes in tRNA modification. Finally, we show that loss of *hTRM9L* in certain tumours can be exploited as a potential chemotherapeutic target since its absence renders tumour cells sensitive to aminoglycoside antibiotics, which induce misincorporation at specific codons leading to protein damage and selective tumour cell killing.

## RESULTS

### Epigenetic silencing of *hTRM9L* in human primary cancers and cancer cell lines

Published evidence and gene expression database mining suggested that *hTRM9L* mRNA is down-regulated in human tumours due to epigenetic gene silencing (Flanagan et al, 2004; Rhodes et al, 2004). To assess the extent of *hTRM9L* mRNA down-regulation in human cancers, we examined a human tumour panel tissue array, covering 18 different cancer types with a total of 306 tumours, for the expression of *hTRM9L* mRNA. We found that *hTRM9L* is significantly down-regulated in testicular, breast and colon cancers followed by cervical and bladder carcinomas (Fig 1B). The tissue array included colon cancer tissue samples ranging from stage I through stage IV. The down-regulation of *hTRM9L* was more pronounced in stage IV cancer, suggesting a progressive loss of *hTRM9L* expression coincided with the acquisition of a more aggressive phenotype and perhaps a later event in progression. We next determined whether *hTRM9L* down-regulation was preserved in colorectal cancer cell lines using quantitative real-time PCR. *hTRM9* transcripts were not detected in three of five cell lines tested, which included HCT116, SW1116 and SW620, while it was present in HT29 and SW480 cells (Fig 1C). However, *hTRM9L* transcript levels were still lower in HT29 cells than in SW480 cells. Thus, colon cancer cell lines were deemed an amenable model to dissect the mechanisms by which *hTRM9L* is turned off and to determine how hTRM9L might affect tumour growth.

We found that treatment of HCT116 or SW620 cells with 5-aza-deoxycytidine (10 µM), which promotes demethylation of 5-methylcytosine to cytosine upon DNA replication, resulted in the restoration of *hTRM9L* mRNA expression (Fig 1D and E). Methylation of tumour suppressors is thought to promote tumourigenesis (Esteller, 2005; Jones & Baylin, 2002). Thus, we tested whether treatment of the highly tumourigenic SW620 cells with 5-aza-deoxycytidine would inhibit tumour growth and whether this correlated with *hTRM9L* re-expression. SW620 cells were cultured in the presence of 5-aza-deoxycytidine (10 µM) for 7 days before being inoculated on chick embryo CAMs to monitor their tumourigenicity (Fig 1F). While control cells produced rapidly growing tumours after 1 week, the 5-aza-deoxycytidine-treated SW620 cells were strongly inhibited in their tumourigenic capacity (*p* = 0.00001). We conclude that *hTRM9L* expression is either directly or indirectly regulated by DNA promoter methylation and that *hTRM9L* expression is inversely correlated with the tumourigenic capacity of SW620 cells. Cumulatively, our data suggests that hTRM9L might have a growth inhibitory/tumour suppressive role in certain cancers.

### hTRM9L-mediated inhibition of colorectal tumour growth is an evolutionary conserved function

**Figure 2:**
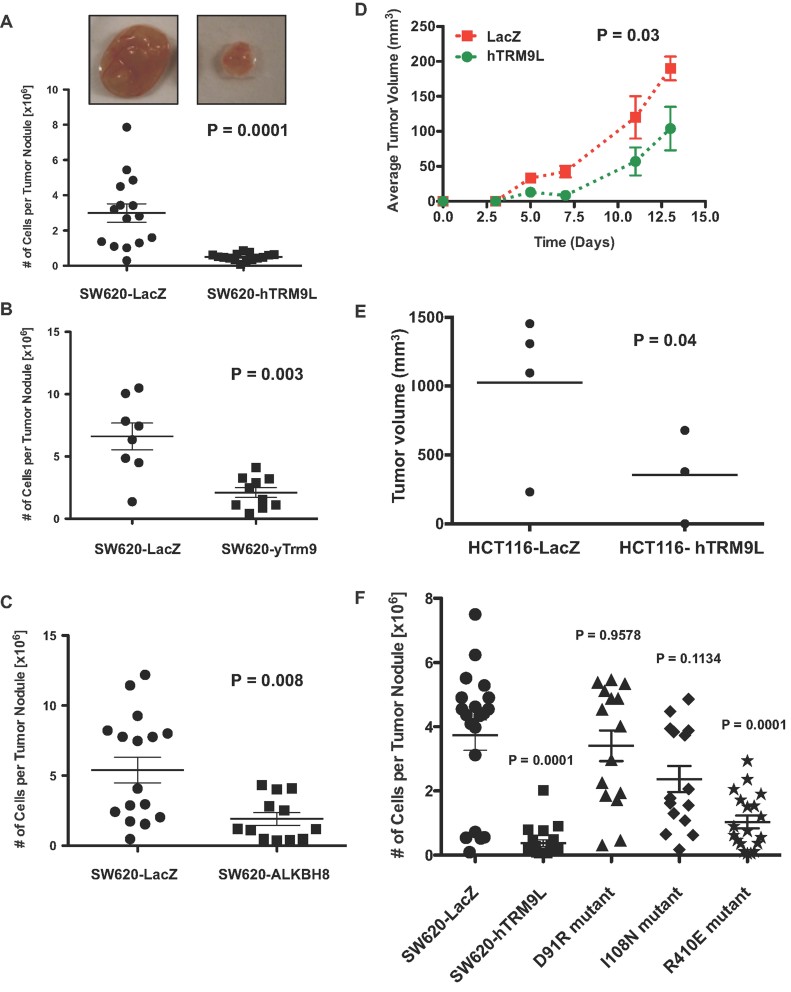
Re-expression of *hTRM9L* and like methyltransferases in SW620 cells inhibits tumour growth *in vivo* **A.** 5 × 10^5^ SW620 cells expressing either *LacZ* or *hTRM9L* were inoculated on CAM and tumours were analysed after 7 days *in vivo*. Tumours were excised, minced and collagenased and the number of cells per tumour nodule was counted.**B,C.** Experiments similar to A were performed with (B) *yTrm9* and (C) mouse *Alkbh8*.**D,E.** SW620 cells expressing either *LacZ* or *hTRM9L* were inoculated in nude mice and tumours were measured every 2 days, up to 11 days. Average data for all days is plotted in (D) and day 11 data for all tumours is shown in (E). HCT116 cells expressing either *LacZ* or *hTRM9L* were inoculated in nude mice and tumours were measured every 2 days, with 12-day data shown.**F.** SW620 cells expressing either LacZ, hTRM9L or its single amino acid mutants hTRM9L-D91R, hTRM9L-I108N and hTRM9L-R410E were inoculated on CAM and tumours were analysed after 7 days. Tumours were excised, minced and collagenased and the number of cells per tumour nodule was counted.

To functionally link *hTRM9L* expression to tumourigenicity, we used a gain of function strategy where SW620 and HCT116 cells were engineered to over-express *hTRM9L*. Because the tRNA methyltransferase activity of Trm9 is conserved from yeast to mammals, we also engineered SW620 cells with the yeast *TRM9* gene (yTrm9) and with mouse *Alkbh8*. Both the cancer cells and the respective controls expressing *LacZ* were tested for tumourigenic capacity in the CAM (SW620) or nude mice (SW620 and HCT116) systems. In contrast to parental, *LacZ*- or *GFP*-expressing SW620 cells, re-expression of *hTRM9L, yTrm9* or *Alkbh8* caused a dramatic inhibition of SW620 tumour growth on CAMs (Fig 2A–C). Injection of *hTRM9L*-expressing SW620 or HCT116 cells in the subcutaneous tissue of nude mice showed that re-expression of this gene resulted in a strong inhibition of tumour growth. In SW620, relative to *LacZ*, *hTRM9L* expressing cells had an extended latency that lasted for an additional week (Fig 2D) and these cells slowed down their growth rate after tumour take. This inhibition of tumour growth was reflected in a significant reduction in the final tumour mass volume compared to control *LacZ* cells (Supporting Information Fig S1A) and this was not only observed for SW620 cells but also when comparing HCT116-LacZ *versus* -hTRM9L cells (Fig 2E). We further used the SW620 model to test whether restored growth of *hTRM9*-expressing cells was due to loss of the transgene. To this end we tested for *hTRM9L* expression in the tumours that grew *in vivo* and we found no correlation between the presence of *hTRM9L* and the growth rate and size of the tumours that grew in the hTRM9L group (Supporting Information Fig S1B). Thus, other mechanisms might allow SW620 cells to eventually bypass the hTRM9L inhibitory function.

To assess whether the methyltransferase activity predicted for hTRM9L was required for the inhibition of tumour growth, we individually mutated two amino acids (D91R and I108N) found in hTRM9L's evolutionarily conserved methyltransferase domain (Kalhor & Clarke, 2003; Katz et al, 2003). Due to their interaction with and use of SAM as a co-factor, most methyltransferase enzymes share this conserved domain structure consisting of three motifs (I-III) that bind and position SAM near the active site. hTRM9L, ALKBH8 enzymes and yeast Trm9 have all been reported to contain these conserved methyltransferase motifs (Kalhor & Clarke, 2003; Songe-Moller et al, 2010) and each enzyme has identical amino acids at positions 91 and 108. We also mutated an amino acid in the unassigned C-terminal region (R410E) of hTRM9L (Supporting Information Fig S1C). The resulting hTRM9L mutants (D91R, I108N and R410E) were then assessed on CAM-based xenografts, relative to SW620 cells expressing *LacZ* or wild-type *hTRM9L*. Mutations to the SAM binding domain (D91R and I108N) abolished the *hTRM9L* dependent growth suppression phenotype, while the R410E mutation outside the SAM binding domain had little effect (Fig 2F). Together the structure–function results support the idea that SAM binding and methyltransferase activity specific to hTRM9L is required for inhibition of tumour growth. Ultimately, our data reveal a previously unrecognized and unanticipated growth-inhibitory function of the methyltransferase activity of hTRM9L.

### hTRM9L induces tumour growth inhibition via a senescence-like arrest

**Figure 3:**
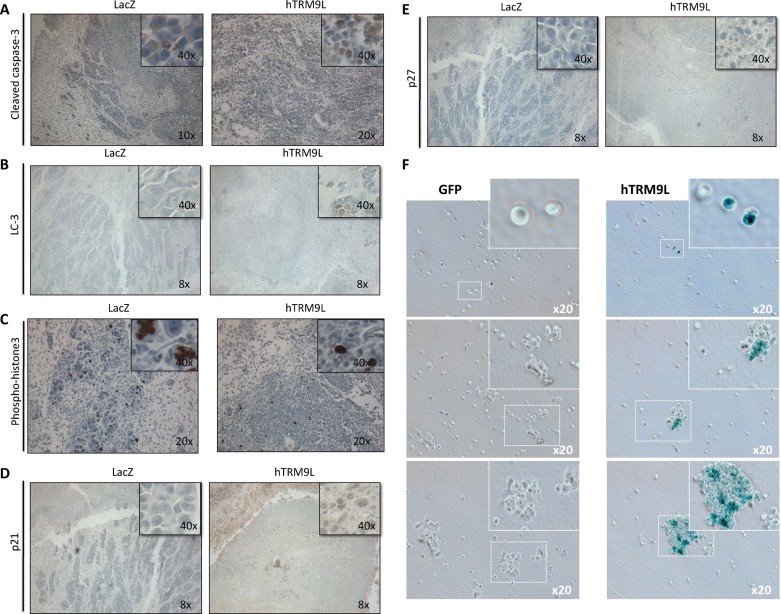
*hTRM9L* dependent growth suppression is associated with increased cellular senescence **A–E.** Tumour nodules of SW620 parent, *LacZ* and *hTRM9L* expressing cells were harvested 7 days post-inoculation, fixed and prepared for (A) cleaved caspase 3, (B) LC-3 (C) phospho-Histone3 (pH3), (D) p21 and (E) p27 immunohistochemistry.**F.** SW620-GFP and *hTRM9L* expressing cells from tumour nodules were harvested after several time points as indicated and stained for acidic β-gal. Statistical significance determined by paired student's *t*-test.

We next explored whether the growth inhibition caused by hTRM9L was due to the induction of apoptosis, autophagic cell death and/or to a growth arrest-associated phenotype. Tumour nodules produced by SW620-LacZ or SW620-hTRM9L cells after a week *in vivo* were sectioned and stained for cleaved caspase-3 (Fig 3A), an apoptosis marker. This staining revealed no major difference in caspase-3 levels, which were overall <7.5%, in the *hTRM9L* positive or negative tumours (Supporting Information Fig S2A). This suggests that the induction of apoptosis is not a major pathway by which *hTRM9L* expressing cells promote growth inhibition. Staining for LC3-I/II to detect any evidence of autophagic cell death also revealed low basal levels of signal (<5%) that did not differ significantly between SW620-LacZ and SW620-hTRM9L tumour nodules (Fig 3B and Supporting Information Fig S2B). These findings suggest that hTRM9L is not inducing gross changes in apoptotic or autophagic cell death that would account for the growth inhibitory function.

The detection of proliferation markers revealed that *hTRM9L* expression in SW620 cells caused a marked decrease in histone-3 phosphorylation (*p* = 0.05) at Ser-10 (Fig 3C and Supporting Information Fig S2C) a residue that is modified during G2-M transition and reveals active transition through this phase. These results support that SW620-LacZ cells are actively proliferating, while SW620-hTRM9L cells are proliferating slowly or not at all. We also detected a consistent up-regulation of the cell cycle inhibitor p21, (*p* = 0.01, Fig 3D and Supporting Information Fig S2D), but found no change in p27 expression in *hTRM9L* tumour nodules (Fig 3E). The combined effect of p21 induction and reduced P-H3 levels suggests that hTRM9L is inducing a growth arrest phenotype. We next monitored senescence-associated β-galactosidase (SA-β-Gal) activity, heterochromatin protein 1γ (HP1γ expression and presence of histone-3-tri-methyl-Lysine-9 marks, all senescence markers (Narita et al, 2006; Sang et al, 2008; Xue et al, 2007; Zhang et al, 2007), in SW620-Control (GFP) or SW620-hTRM9L tumour nodules or after their digestion using collagenase-I. In these experiments, independent batches of control (GFP) or *hTRM9L* expressing cells were generated to allow for β-galactosidase activity measurements. We analysed β-galactosidase activity derived from either SW620-Control (GFP) or SW620-hTRM9 in 1-week old tumour nodules (Fig 3F) and found that there was a strong up-regulation of SA-β-Gal activity in SW620-hTRM9L cells, relative to SW620-GFP, with 30% of the hTRM9L proficient and 5% of the control cells expressing SA-β-Gal, respectively. Although some inter-experiment variability was observed for the absolute levels of senescence, *hTRM9L*-expressing tumour nodules were consistently higher in SA-β-Gal activity and peaked 3 days after *in vivo* inoculation. Close to 80% of the *hTRM9L* proficient cells stained positive for SA-β-Gal activity after 3 days and up to 7 days *in vivo*, with a maximum of 40% of the control cells staining positive for SA-β-Gal activity 5-days post inoculation (Supporting Information Fig S2E). However, we detected no difference in HP1γ staining, that displayed a nuclear signal in both SW620-GFP or SW620-hTRM9L cells in tumour histological sections (Supporting Information Fig S2F). We also detected very low levels of H3K9me3 marks in these tumour sections when comparing the same cells lines (Supporting Information Fig S2F). Our results reveal that hTRM9L-induced tumour growth inhibition in SW620 cells is not due to increased apoptosis or autophagy; rather it is caused by a growth arrest phenotype that only partially recapitulates the expression of key markers of a canonical senescence phenotype (Collado & Serrano, 2006; Narita et al, 2006; Xue et al, 2007). Thus, hTRM9L is able to induce a “senescence-like phenotype” associated with SA-β-gal activity and p21 expression up-regulation that is strongly associated with a G0/G1 arrest.

### hTRM9L-dependent up-regulation of the tumour suppressor *LIN9* is linked to the growth inhibitory phenotype

**Figure 4:**
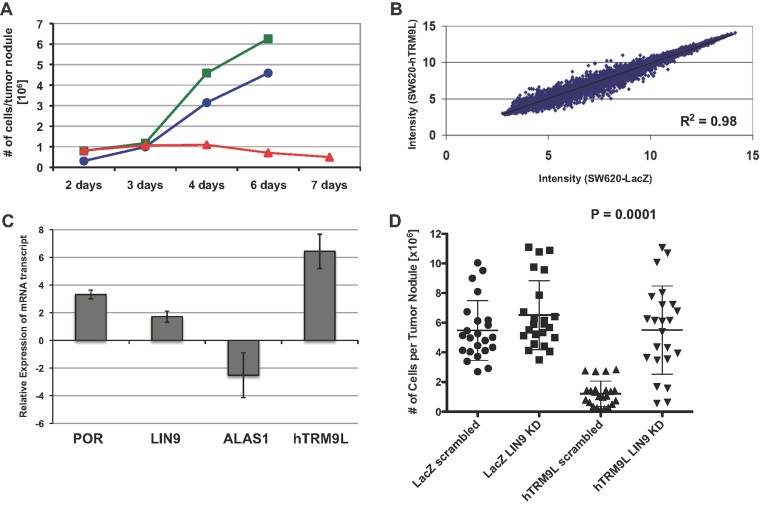
*LIN9* transcripts are up-regulated in *hTRM9L* expressing cells and are responsible for the growth suppression phenotype SW620 (blue circle), *LacZ* (green square) and *hTRM9L* (red triangle) tumours were harvested after several time points as indicated, minced, collagenased and quantitated as before.The intensity values measured by microarray analysis are represented in a scatter plot comparing expression patterns of SW620-LacZ (*x*-axis) and SW620-hTRM9L (*y*-axis) of 3-day-old tumours grown on the CAM. Each gene in the microarray is represented by a dot with coordinates consisting of average gene expression measured from three different replicates of RNA samples. A linear regression line is overlaid with the scatter plot and the regression equation is displayed. Outliers represent up-regulated or down-regulated genes.Real-time PCR validation of *POR, LIN9, ALAS1*, and *hTRM9L* respective expression levels. RNA was isolated from a GFP-positive population isolated from 3 day tumours grown on the CAM and amplified using MessageBOOSTER™ cDNA Synthesis Kit. Relative quantification is presented after normalization with GAPDH.Cells with a knock down of *LIN9* or a scrambled control, in *hTRM9L* and *LacZ* expressing variants, were assayed for their tumour forming capacity, along with the parental cells. Statistical significance determined by paired student's *t*-test.

The fact that SW620 cells are derived from a metastatic lesion and that SW480 cells are derived from the primary tumour from the same patient opened the possibility that hTRM9L might affect metastatic capacity related to invasion and dissemination. However, we did not find any clear indication that hTRM9L affects intravasation or dissemination to the liver in the chick embryo metastasis assay (Supporting Information Fig S3A and S3B), suggesting that loss of *hTRM9L* in the SW620 metastatic variant is not related to gain of invasion and dissemination properties in these cells. Time course analysis of CAM *in vivo* tumour growth revealed that 3 days after inoculation, the *hTRM9L* deficient cells begin to proliferate while the *hTRM9L* proficient cells are already in the senescence-like arrest (Fig 4A). Thus, within 24–48 h after injection, SW620-hTRM9L cells are unable to mount a strong proliferative behaviour and at day 3 this phenotypic discrepancy is significant. We performed gene expression profiling of SW620-LacZ and SW620-hTRM9L using the Affymetrix platform, to identify the signalling pathways affected by hTRM9L. We took advantage of the GFP tagging present in SW620 and sorted cells from 3-day-old tumour nodules using FACS, as this is when SW620-LacZ cells begin actively proliferating and SW620-hTRM9L cells are arrested. This strategy revealed significant changes in gene expression in only a small subset of genes that were up-regulated (25-transcripts) or down-regulated (37-transcripts) in *hTRM9L* expressing cells, relative to *LacZ* (Fig 4B, Table 1). The magnitude of gene expression change was not larger than threefold (except for *hTRM9L* in the SW620-hTRM9L cells). We validated these findings for a subset of transcripts to confirm the increase levels for *POR, LIN9* and *hTRM9L* and decreased *ALAS1* levels in *hTRM9L*-expressing cells (Fig 4C). Thus, it appears that only a limited number of genes are regulated at the transcriptional level by hTRM9L, and that they need not change dramatically in transcriptional abundance to associate with a strong growth inhibition.

**Table 1:** Transcripts regulated in SW620-hTRM9L *versus* SW620-LacZ cells (log_2_-based fold change)

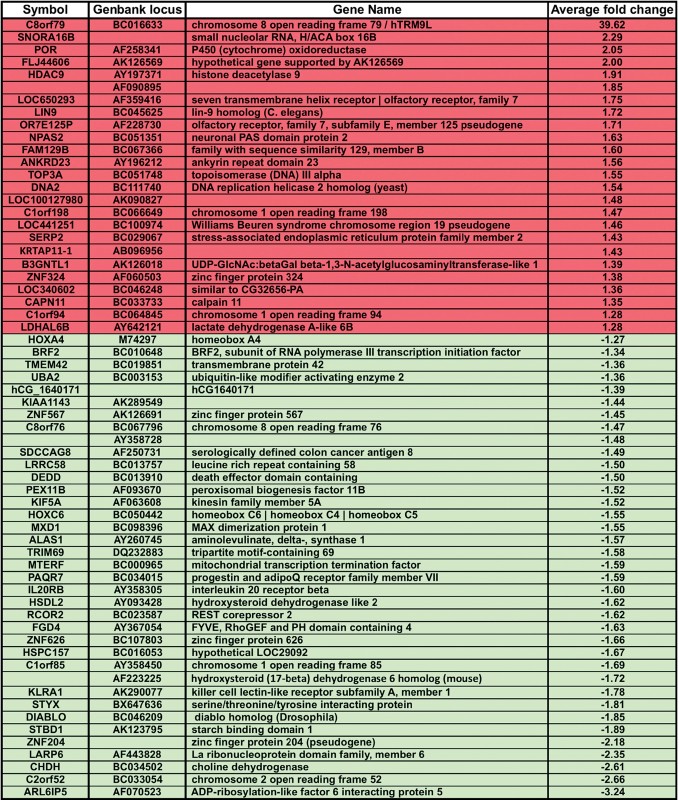

Among the genes induced by hTRM9L, we identified the tumour suppressor *LIN9*, a mammalian homolog of the *C. elegans* LIN9 protein that is part of the transcriptional repressor DREAM complex (also known as the LINC complex) (Pilkinton et al, 2007a; Sandoval et al, 2009). We validated that the *LIN9* transcript and protein is up-regulated ∼2-fold in *hTRM9* expressing cells (Supporting Information Fig S3C) and reasoned that this could be one of the pathways used by such cells to induce growth arrest in xenografts. LIN9 has been demonstrated to act as a tumour suppressor itself as it can bind to pRB (Gagrica et al, 2004). We reasoned that if increased *LIN9* expression in the *hTRM9L* expressing cells was responsible for the corresponding growth suppressive phenotypes in xenografts, then a knockdown of *LIN9* in *hTRM9L* expressing cells should restore the proliferative phenotype associated with SW620 parental or *LacZ* expressing cells. Using specific shRNAs, we obtained ∼93% knockdown of *LIN9* in *hTRM9L* expressing SW620 cells (Supporting Information Table 1). We inoculated the parental and *LIN9* knockdown cells and analysed for tumour formation on the CAM at 7 days. We found that knockdown of *LIN9* completely reverses the hTRM9L phenotype and significantly increases the proliferation of SW620-hTRM9L expressing cells (*p* = 0.0001), relative to a scrambled knockdown (Fig 4D). Knockdown of *LIN9* in SW620-lacZ cells did not show any significant effect on cell proliferation. Our experiment results associated with LIN9 loss of function in *hTRM9L* expressing SW620 cells, clearly demonstrate that part of the mechanism of tumour growth inhibition involves a known component of the DREAM complex and cell cycle machinery.

### hTRM9L expression prevents tumour growth under hypoxic conditions in SW620 cells

**Figure 5:**
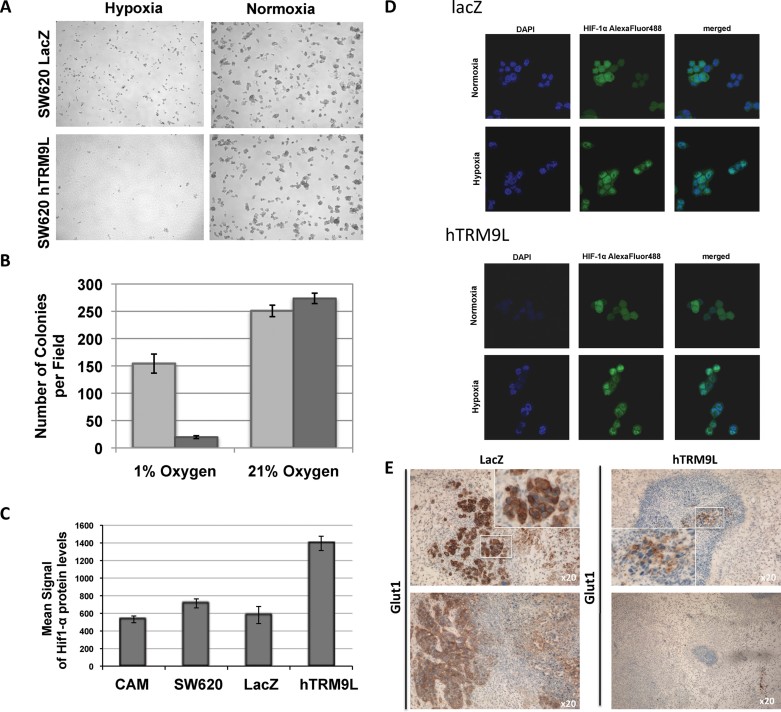
Hypoxic growth prevented in *hTRM9L* re-expressing cells **A,B.** SW620-LacZ (light grey bars) and SW620-hTRM9L (dark grey bars) expressing cells were grown under 21% oxygen (normoxic) and 1% oxygen (hypoxic) conditions and the colonies formed were quantitated. No apparent growth difference on the cell lines was detected when grown under normal oxygen conditions, whereas exposure to hypoxia revealed a growth-sensitive phenotype of *hTRM9L* expressing cells.**C.** Protein lysates prepared from 3-day-old tumours were added to MSD MULTI-SPOT 4-Spot plates coated with anti-total-HIF1-α antibody. Total HIF1-α was detected with anti-total-HIF1-α antibody labeled with MSD SULFO-TAG reagent. *hTRM9L* expressing tumour cells show a twofold increase in HIF1-α protein levels compared to control tumour cells.**D.** Immunofluorescence microscopy analysis of HIF1-α in hTRM9L deficient and proficient cells under normoxic and hypoxic conditions. Images were not fixed in exposure time, thus, they do not reflect the quantitative protein abundance difference shown in (C).**E.** Levels of downstream markers for HIF1-α target activation, GLUT1, were analysed in both cells types.

The tumour microenvironment can include multiple cellular stresses, with hypoxia being an underlying theme. We postulated that adaptation to low oxygen levels might be one of the triggers associated with the hTRM9-dependent growth suppression. To test our hypothesis, we cultured SW620-LacZ and SW620-hTRM9L cells under normoxic (21% O_2_) and hypoxic (1% O_2_) conditions for 12 days. We determined that the number of colonies derived from SW620-hTRM9L expressing cells was significantly reduced when cells were cultured under hypoxic conditions, relative to SW620-LacZ (Fig 5A and B). This result was in contrast to the observation that both cell types can form similar numbers of colonies under normoxic conditions. A key response pathway operating under hypoxic conditions is controlled by HIF1-α, a transcription factor that is stabilized and targeted to the nucleus in response to decreased O_2_ levels (Lisy & Peet, 2008; Yee Koh et al, 2008). In our CAM xenograft model, we tested whether the HIF1-α pathway was perturbed in *hTRM9L* expressing cells, relative to SW620-LacZ. First, we performed a quantitative analysis of HIF1-α protein levels in SW620, SW620-LacZ and SW620-hTRM9L expressing cells grown 3 days post inoculation on the CAM (Fig 5C). We determined that HIF1-α protein levels were ∼2-fold higher in *hTRM9L* expressing cells, relative to LacZ, a finding that was surprising as these cells fail to grow under hypoxic conditions or on xenografts. The increased HIF1-α levels indicated that the initial response to hypoxic conditions is mounted in *hTRM9L* expressing cells but that it might be over-compensating because downstream signals are not activated. We monitored HIF1-α nuclear localization under hypoxic conditions and determined that nuclear localization was occurring at similar levels (∼65%) in both *hTRM9L* and *LacZ* expressing cells (Fig 5D). These data suggest that the ability of HIF1-α to become translocated into the nucleus upon hypoxia is not a limitation. To determine if nuclear localized HIF1-α was somehow inactive in *hTRM9L* expressing cells, we used immunohistochemistry to monitor the *in vivo* expression of GLUT1, a prototypical hypoxia and HIF1-α target gene (Chen et al, 2001). In each case, we counted ∼400 stained SW620 cells expressing *LacZ* or *hTRM9L* and observed a highly significant difference (*p* = 0.0009) in GLUT1 expression. We found that while control SW620-LacZ cells were able to express high levels of GLUT1 (∼75%) that clearly localized to the plasma membrane, SW620-hTRM9 tumour sections showed a marked decrease in GLUT1 expression (∼5%) that was irregularly distributed in a membrane-like pattern (Fig 5E). There was a significant difference (*p* = 0.0009) in the number of GLUT1 positive cells when we compared SW620-*LacZ* to those expressing *hTRM9L* (Supporting Information Fig S4). We conclude that while HIF1-α appears to be responsive to O_2_ tensions, this does not correlate with efficient GLUT1 protein accumulation, which is required to adapt to the demands of a hypoxic microenvironment.

### Loss of hTRM9L in human tumours allows for selective killing of cancer cells with the translational error inducer paromomycin

**Figure 6:**
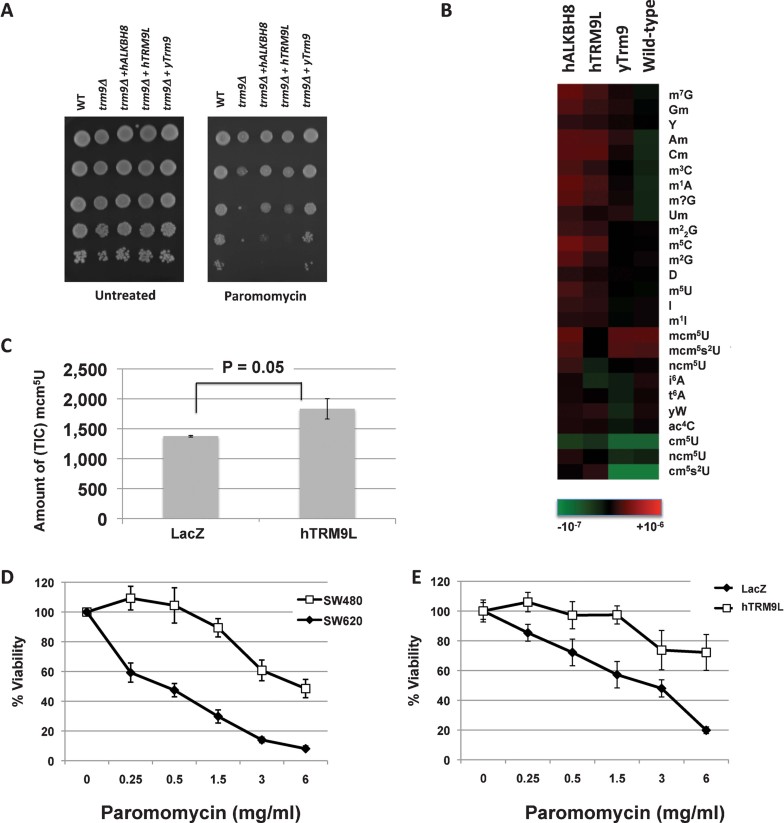
hTRM9L affects tRNA modification and prevents aminoglycoside antibiotic induced death Complementation analysis of *trm9*Δ yeast cells with *hTRM9L*, *hALKBH8* and *yTrm9*.Hierarchical clustering of tRNA modification data, with significance calculated relative to *trm9*Δ cells. tRNA modification increases were assigned a positive *p*-value, while decreases were assigned a negative *p*-value.SW620-lacZ and SW620-hTRM9L cells were left untreated or exposed to 1.5 mg/ml paromomycin for 24 h. For data reported in (B) and (C), small RNA was isolated and validated to be of high integrity by Bioanalyser analysis. RNA was enzymatically digested to nucleosides and the identity and levels, as determined by total ion count (TIC), of specific modified nucleosides was determined by LS/MS-MS analysis.SW480 and SW620 cells were treated with paromomycin and the percent viability for each was determined by trypan blue staining 24 h post exposure.SW620-LacZ and SW620-hTRM9L cells were exposed to paromomycin and assays were performed as described above.

A deficiency in yeast Trm9 renders cells sensitive to the aminoglycoside antibiotic paromomycin (Kalhor & Clarke, 2003). Complementation analysis demonstrated that re-expression of either *hTRM9L*, human *ALKBH8*, or *yTrm9* in *trm9*Δ *S. cerevisiae* cells could rescue the paromomycin sensitive phenotype (Fig 6A). We note that ALKBH8 provided more rescue then hTRM9L, with neither activity rescuing to levels demonstrated by yTrm9. Next using these yeast systems, we performed quantitative LC-MS/MS analysis on purified tRNA derived from wild-type, *trm9*Δ and *trm9*Δ cells expressing either *yTRM9, hALKBH8* or *hTRM9L*. The *trm9*Δ yeast represents a ideal model as these mutants have been shown to be devoid of mcm^5^U and mcm^5^s^2^U modifications (Chan et al, 2010) while providing the yTrm9 substrate cm^5^U, which we have confirmed (Supporting Information Tables 2 and 3). We quantified the levels of 28 modifications, which included intermediaries in the mcm^5^U and mcm^5^s^2^U biosynthetic pathways, as well as 22 other tRNA modifications. The mcm^5^U and mcm^5^s^2^U modifications were only identified in wild-type, *trm9*Δ + *yTrm9* and *trm9*Δ + *hALKBH8* cells, with hALKBH8 only being able to generate a fraction of what we observed for wild-type cells. Notably, we observed a significant decrease in the levels of the cm^5^U substrate in both *hALKBH8* (*p* = 0.002) and *hTRM9L* (*p* = 0.006) expressing cells, relative to the *trm9*Δ cells, supporting the idea that both of these activities affect this yTrm9 substrate. Surprisingly, in *hTRM9L*-expressing cells we observed significant increases in the levels of 11 other tRNA modifications (Am, Cm, Gm, I, m^1^A, m^2^_2_G, m^2^G, m^3^C, m^5^C, m^7^G and yW), relative to *trm9*Δ cells, with 10 of the 11 also significantly up-regulated in hALKBH8-expressing cells. Our data suggest that both mcm^5^U/mcm^5^s^2^U and global reprogramming of tRNA modifications provide paromomycin resistance in yeast, which is a novel finding.

Next, we postulated that *hTRM9L* re-expression would perhaps have a similar effect on human cells and promote a change in tRNA modification. Further as ALKBH8 is present in SW620 cells, the presence of hTRM9L may promote an increase in mcm^5^U levels. Based on the yeast rescue data these changes should be prominent under translational stress conditions. We performed quantitative LC-MS/MS analysis on purified tRNA derived from SW620-LacZ and SW620-hTRM9L cells left untreated or exposed to paromomycin. We quantified the levels of 26 tRNA modifications from the four sample types (Supporting Information Tables 4 and 5). Under untreated conditions, we observed a significant increase in 10 tRNA modifications (ac^4^C, Cm, Gm, I, i^6^A, m^1^A, m^2^G, m^5^Um, OHyW and t^6^A) in *hTRM9L*-expressing cells, relative to *LacZ*. We also observed a significant increase in the levels of 4 tRNA modifications (ac^4^C, m^1^A, mcm^5^U, t^6^A) in *hTRM9L*-expressing cells after paromomycin treatment, relative to paromomycin treated SW620-LacZ. Paromomycin treatment leads to a significant decrease (*p* = 0.001) in mcm^5^U (the ALKBH8 specific modification) levels in SW620-LacZ cells. In contrast, there was a 20% increase (*p* = 0.05) in mcm^5^U modifications in SW620-hTRM9L expressing cells after paromomycin treatment, relative to untreated (Fig 6B). Our functional analysis supports the idea that hTRM9L can promote an increase in mcm^5^U levels and other tRNA modifications, but the former might be more prominent under translational stress. This suggested that the loss of hTRM9L might render tumours less adaptable to drugs that induce translational stress and perhaps drugs like paromomycin might selectively affect tumours where *hTRM9* is silenced.

To test this possibility, we next determined whether a deficiency in hTRM9L was associated with sensitivity to paromomycin in human cells. To initially make this comparison, we used SW620 metastatic (*hTRM9L*-) and SW480 primary tumour (*hTRM9L+*) cells that are derived from the same patient providing a relatively isogenic system. When SW620 cells (*hTRM9L*−) and SW480 cells (*hTRM9L+*) were tested for sensitivity to paromomycin (Fig 6C), we determined that the *hTRM9L* deficient cells (SW620) had decreased viability relative to their *hTRM9* proficient counterparts (SW480). We also found that re-expression of *hTRM9L* in SW620 cells rescued the sensitivity to this aminoglycoside antibiotic (Fig 6D). There appears to be a low threshold for sensitivity to paromomycin in hTRM9L-deficient cells. Further, there is little difference in paromomycin sensitivity when comparing SW480 to SW620-hTRM9L expressing cells (Supporting Information Fig 5). In the SW620 background, the sensitivity of hTRM9L deficient, *versus* proficient, cells was also observed using the aminoglycoside antibiotic gentamicin (Supporting Information Fig 6). In addition, HT29 and HCT116 cells expressing hTRM9L were resistant to paromomycin, relative to LacZ expressing cells (Supporting Information Fig 6B and C). We conclude that a lack of hTRM9L, and possibly due to the inability of cells to modulate the levels mcm^5^U and other tRNA modifications, may represent a deficiency in tumours that might be specifically exploited to induce killing of these tumour cells.

## DISCUSSION

**Figure 7:**
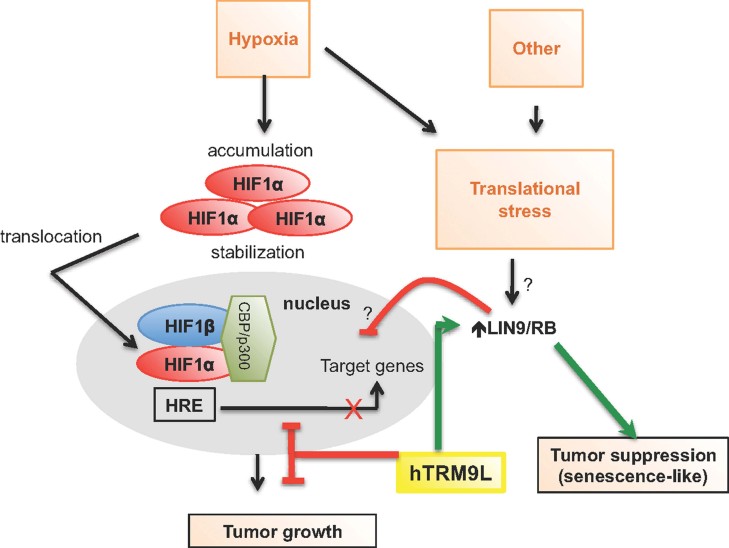
Proposed mechanism of growth suppression in hTRM9L proficient cells Direct or indirect regulation of *LIN9* and the hypoxic response lead to decreased tumour growth in hTRM9L positive cells.

Our study reveals a previously unrecognized function for the *hTRM9L* gene in regulating stress adaptation and tumour growth. We propose the following working model to account for hTRM9L's influence on cancer cell proliferation (Fig 7). hTRM9L, via its methyltransferase activity, likely influences the translation of a specific protein or proteins involved in the regulation of *LIN9*. While there are only a few published reports that give attention to LIN9 (Gagrica et al, 2004; Osterloh et al, 2007; Pilkinton et al, 2007a, b; Reichert et al, 2010; Sandoval et al, 2009), there is a clear connection between LIN9 and cell cycle regulation. In our system, activation of *LIN9* in *hTRM9L* expressing cells is connected to the onset of a G0–G1 arrest and a senescence-like phenotype, which accounts for the non-proliferative phenotype. That not all markers of senescence were induced by hTRM9L expression is not unexpected as senescence is a restrictive mechanism that is evaded early during tumour progression (Beausejour et al, 2003; Narita et al, 2006; Xue et al, 2007). One possible explanation is that hTRM9L-dependent induction of *LIN9*, in addition to inhibiting exit from G1, short circuits the response to hypoxia and prevents cells from thriving in the oxygen poor tumour microenvironment. As part of our study, we have identified a disconnect between the translocation of HIF1-α into the nucleus and the transcriptional regulation of corresponding targets (*i.e.* GLUT1). It is possible that in the *hTRM9L* cells LIN9 up-regulation could directly or indirectly interfere with HIF1-α to prevent transcription of downstream genes.

Modifications of tRNA bases can provide an additional level of gene regulation and have been shown to regulate the levels of specific proteins. Yeast Trm9 enhances the translation and levels of the AGA and GAA codon rich transcripts corresponding to the ribonucleotide reductase large subunits to promote an efficient response to DNA damage (Begley et al, 2007). Klungland and coworkers have provided evidence that mouse Alkbh8 and the catalysed mcm^5^U wobble base modifications are vital for the translation of the reactive oxygen species detoxifying protein Gpx1 (Songe-Moller et al, 2010). Optimization of Gpx1 levels appears to be working by stop codon recoding to selenocysteine.

The protein sequence for hTRM9L, structure-function results as well as the complementation and tRNA modification analysis data support that the corresponding activity can affect tRNA modification. Clearly though, the hTRM9L-associated paromomycin rescue of *trm9*Δ yeast cells is not due to the formation of mcm^5^U and mcm^5^s^2^U and appears to be due to some global reprogramming of tRNA modifications. In addition, our yeast data supports the idea that ALKBH8 is the main tRNA methyltransferase used for mcm^5^U and mcm^5^s^2^U formation. While the exact biochemical activity of hTRM9L is still in question, our yeast tRNA modification data does demonstrate significant changes to many methyl-based modifications as well as a decrease in the cm^5^U substrate. Our global tRNA modification data in mammalian cancer cells also demonstrates that under basal conditions the SW620 *hTRM9L* expressing cancer cells have a distinct difference in the levels of 11 tRNA modifications, relative to SW620-LacZ that we have linked to a growth suppressive signal in cancer cells. Our paromomycin treatment results in SW620 cells further implicates wobble base modifications as being vital to the response. Notably, paromomycin treatment leads to a significant reduction in mcm^5^U, as well as to an increase in m^5^C and I modifications, in SW620-LacZ expressing cells, relative to untreated cells. These paromomycin-induced changes in tRNA modification were counteracted in SW620-hTRM9L expressing cells. Importantly, in SW620-hTRM9L cells there was increased mcm^5^U levels after paromomycin treatment, compared to treated SW620-LacZ, supporting a role for hTRM9L in wobble base modification. This along with results from our structure-function studies suggests that stress-induced signals activate the methyltransferase activity of hTRM9L. Further experimentation is clearly needed to clarify the precise biochemical activity of hTRM9L. The absence of mcm^5^U and mcm^5^s^2^U modifications in *trm9*Δ cells expressing hTRM9L, suggests that hTRM9L catalyses the formation of another modification, works on specific tRNA to generate mcm^5^U modifications, regulates other tRNA methyltransferases or requires activation or cofactors present in mammalian but not in yeast cells. Identifying the specific biochemical activity and tRNA substrates as well as the targets of translational regulation linked to hTRM9L is needed to understand the role that this enzyme plays in tumour growth suppression.

Colorectal cancer is a result of a multistep process derived from cumulative gene alterations arising from genome instability and epigenetic modifications (Ilyas et al, 1999). The hypermethylation of CpG islands in promoter regions of many genes, such as tumour suppressors, that can block transcription initiation and alter chromatin structure results in gene expression changes leading to cancer development (Esteller, 2002). Our study putatively links transcriptional silencing of *hTRM9L* to changes in translational and transcriptional potential that would favour tumour growth. While our experimental model was focused on colorectal cancer, we also observed a significant decrease in *hTRM9L* expression levels in cancers found in the testis, breast, cervix and bladder. Thus, silencing or loss of *hTRM9L* may be a more widespread mechanism that allows cancer cells to progress to a more aggressive state. The finding that hTRM9L results in the induction of *LIN9* suggests that regulation of translation elongation via tRNA modifications is a key control point-regulating exit from G1. Elucidation of the hTRM9L regulatory pathway and of the players involved, as well as their link to the stress-induced suppression of the hypoxic response, might open a previously unrecognized paradigm of rapid signalling from translation elongation to the G1–S transition of the cell cycle.

Knowledge gained from our aminoglycoside-based mechanistic studies identified a potential ‘Achilles’ heel' for hTRM9L deficient tumours, in that they are highly sensitive to drugs that induce translational errors. The absence of hTRM9L leads to a sensitivity to paromomycin, a finding that can be exploited to specifically target hTRM9L^negative/low^ tumour cells. Thus, during progression, a growth advantage gained from *hTRM9L* silencing creates a phenotype that under specific stresses (*i.e.* translational) might become lethal. However, tumours might not encounter this stress in a sufficient degree that it is detrimental for their growth. A similar weakness is found in tumours that rely heavily on AKT signalling from growth factors. Increased AKT signalling is at the expense of ROS detoxifying capacity because of FOXO3a inhibition by AKT (Dolado & Nebreda, 2008; Nogueira et al, 2008). Thus, AKT^high^/FOXO3a^low^ tumours become highly sensitive to ROS inducing drugs. Similarly, because hTRM9L^low^ tumours become sensitive to paromomycin, additional drugs could be used to exploit this weakness in the clinical setting. Aminoglycoside antibiotics are an attractive class of drugs because many of the over 25 varieties are approved by the Food and Drug Administration (FDA) in the treatment of infections by gram-positive bacteria (Brunton et al, 2005). We propose that elucidation of the mechanism by which hTRM9L activity affects translation and paromomycin sensitivity may allow us to exacerbate this translational error phenotype for therapeutic purposes.

## MATERIALS AND METHODS

### Cells, culture conditions and treatments

The human colorectal cancer cell lines SW620, SW480, HT29, HCT116 and SW1116 were purchased from ATCC. SW620, HT29 and HCT116 were cultured in Dulbecco's Modified Eagle's Medium (Hyclone) supplemented with 10% FBS (Sigma), 100 units/ml penicillin and 100 µg/ml streptomycin (Hyclone) at 37°C in 5% CO_2_ humidified air. SW480 and SW1116 cell lines were cultured in Leibovitz's L-15 Medium (ATCC) at 37°C in 100% humidified air. All *in vitro* experiments were performed at 60–70% cell density to avoid the effects of confluence. For epigenetic reprogramming experiments, SW620 cells were treated with 10 µM 5-aza-dC (Sigma–Aldrich) over a period of 7 days with the culture being replaced every 24 h with fresh growth media containing 5-aza-dC. We note that we did not observe any significant difference in cellular proliferation or viability when compared to SW620-LacZ and SW620-hTRM9L expressing lines grown in cell culture. Further, after 5-aza-deoxycytidine treatment, we did not observe any appreciable toxicity in either of the cell lines. For the aminoglycoside antibiotic viability assay, SW620 and SW480 cells were treated with Paromomycin Sulfate or Gentamicin Sulfate (Sigma–Aldrich) for 24 h and viability was determined by the trypan blue exclusion method. Yeast experiments were performed using BY4741 cells. The *KIAA1456, mALKBH8, hALKBH8* and *yTRM9* genes were PCR amplified with 40 base pair overhangs, directed to 40 base pairs upstream and 40 base pairs downstream of the native *TRM9* locus. Each construct was targeted to the native *TRM9* locus by transforming into *trm9*Δ cells, which were generated with a *URA3* deletion cassette. Transformants were selected on 5-FOA plates and targeting was confirmed by PCR. Site directed mutagenesis of hTRM9L was performed using PCR based approaches and verified by DNA sequence analysis. The primers were designed to replace the appropriate bases to change the aspartic acid (GAC) to arginine (AGA) at position 91 (D91R mutant), and isoleucine (ATA) to asparagine (AAT) at position 108 (I108N mutant) and arginine (CGC) to glutamic acid (GAG) at position 410 (R410E mutant). Stable SW620 cells expressing each hTRM9 mutant construct were made by retroviral transduction described below.

### Production of stable cell lines

The *hTRM9L* cDNA sequence was PCR-amplified from a c8orf79 bacterial clone purchased from ATCC, subcloned into a Flag Tag Expression vector (pCMV-3X FLAG) (STRATAGENE); 3X FLAG hTRM9L was then cloned into retroviral expression vector pBabePuro (Addgene). The retroviral expression vector pBabePuro-β-galactosidase was a generous gift from Dr. Conklin (CRC, SUNY Albany). EGFP was PCR-amplified from pMSCV sinpuro-EGFP and cloned into pBabeHygro (Addgene). Each retroviral expression construct was transfected into Phoenix-Ampho packaging cells by using Fugene6 Transfection reagent (Roche Applied Science). After 72 h, the retrovirus-containing supernatant was collected and filtered through a 0.45 µm filter to remove cellular debris. SW620 cells were infected with the viral supernatant/polybrene mixture (8 µg polybrene per millilitre viral supernatant). Three days post-infection, the infected cells were selected for using puromycin (2.5 µg/ml, MP Biochem.) and hygromycin (800 µg/ml, Roche) for 7 days, after which stable cell lines were expanded. HCT116 and HT29 lines expressing hTRM9L or LacZ were constructed as described above. *LIN9* knockdowns in SW620 *LacZ* and *hTRM9L* expressing cells were achieved by stable retroviral infections. pRS shRNA expression vectors against *LIN9* and scrambled control were purchased from Origene. Retroviruses (pRS/HuSH Lin9 and pRS/HuSH scrambled) were produced in Phoenix-Ampho packaging cells and cell lines were first infected with the viral supernatant and then selected with the appropriate antibiotic as described above.

### Real-time PCR analyses

For real-time PCR, the total RNA was isolated using Trizol Reagent (Invitrogen) and subsequently purified by ethanol precipitation. Quantitative TaqMan PCR analysis was carried out with the ABI PRISM 7900HT Sequence Detection System (Applied Biosystems) using a TaqMan One-Step RT-PCR Master Mix Reagents Kit (Applied Biosystems) in a reaction volume of 20 µl containing 2 µg purified RNA, 1× Master Mix without UNG, 1× MultiScribe and RNase Inhibitor Mix and 1× probes and primer sets Hs00332747_m1 (KIAA1456) (TaqMan Gene Expression Assays) or 1× HuGAPDH (Pre-Developed TaqMan Assay Reagents) according to manufacturer's protocol. The following Thermal Cycler parameters were used: incubation at 48°C for 30 min (RT-step), denaturation at 95°C for 10 min, followed by 40 cycles of the amplification step (denaturation at 95°C for 15 s and annealing/extension at 60°C for 1 min). All amplification reactions were performed in triplicate and the relative quantification of *KIAA1456* (*hTRM9L*) gene expression was determined after normalization with the endogenous control, *GAPDH*. Data processing and statistical analyses were performed using the ABI PRISM SDS, software version 2.1 (Applied Biosystems). All mRNA data points specific to cancer lines and xenograft tumour samples represent the average of independent biological samples (*N* = 3), with error bars representing standard deviations and statistical significance determined using a Student's *t*-test.

### TissueScan human cancer panel

*KIAA1456* (*hTRM9L*) expression profiling was performed using TissueScan Cancer qPCR arrays (384 format) from OriGene (OriGene Technologies, Inc., Rockville, MD, USA). The qPCR array consisted of panels of normalized first-strand complementary DNA (cDNA) from 18 different pathologist-verified human cancer tissues (with cancer stage ranging between I to IV). The array also included samples derived from normal tissues. Quantitative PCR analysis was carried out with the ABI PRISM 7900HT System (Applied Biosystems) and the expression level of *hTRM9L* in these cancer tissues was examined using the primer set Hs00332747_m1 (KIAA1456/hTRM9L) (TaqMan Gene Expression Assays) after normalization with the control gene SybrGreen β-actin (Origene-supplied primer pair) according to manufacturer's protocol. For the tumour array panel we used technical replicates (*N* = 3) of each patient sample to generate average values. The average value from each tumour sample in a cancer subtype for each patient was compared to matched normal tissue using a Student's *t*-test.

### Xenografts: chick chorioallantoic assay (CAM Assay) and nude mice

Fertilized White Leghorn chicken eggs (Charles River, MA) were incubated for 10 days at 37°C in a humidified atmosphere inside a hatching incubator equipped with an automatic rotator (Octagon 20, Brinsea, Somerset, UK). On the day of the experiment, each eggshell was punctured in two locations: one on the long side of the egg and one on the side over the natural air sac. By using a suction device, an artificial air sac was created to separate the CAM from the eggshell. A square window of ∼1 cm was opened over the displaced CAM and sealed with a piece of sterile tape. GFP labeled SW620 cells expressing β-galactosidase or hTRM9L were detached from the plate with 2 mM EDTA in PBS and washed twice in PBS. Cells (5 × 10^5^) were resuspended in 50 µl PBS containing 1 mM MgCl_2_, 0.5 mM CaCl_2_, 100 U/ml penicillin and 100 µg/ml streptomycin and then inoculated per CAM. The opening was re-sealed with tape and the eggs were placed in a stationary incubator at 37°C for 7 days or as indicated. The resulting tumours were excised and minced in a clean petri dish, then collagenased (type IA, Sigma) for 30 min at 37°C. The number of tumour cells was counted with a haemacytometer. For nude mouse studies, SW620-LacZ, SW620-hTRM9L, HCT116-LacZ and HCT116-hTRM9L cells were detached with 2 mM EDTA, counted, and 3.75 × 10^5^ cells in 100 µl of PBS were injected subcutaneously into the interscapular and flank regions of 4- to 6-week-old BALB/c nu/nu mice (Charles River, Wilmington, MA). Tumours were measured every 2 days or upon completion of the experiment. Tumour volumes were calculated using the formula (*D* × *d*2)/2, where *D* is the longest and *d* is the shortest diameter. Animals were sacrificed at 7 weeks. Tumours were flash frozen for transgene detection or fixed in 10% formalin and embedded in paraffin. All data points represent independent biological samples (*N* = 3), with error bars representing standard deviations and statistical significance determined using a Mann–Whitney test.

### Detection of disseminated SW620 cells via Alu-qPCR

SW620 overexpressing hTRM9 or LacZ cells were inoculated into CAMs (for details see (Kim et al, 1998) and allowed to grow for 3 days. Livers were collected and incubated with collagenase. DNA was extracted using the Extract-N-Amp kit from Sigma (XNAT2-1KT). Human Alu (hAlu) sequences were detected by quantitative-PCR. The primers for hAlu sequences were: hAlu sense (ACGCCTGTAATCCCAGCACTT) and hAlu antisense (TCGCCCAGGCTGGGTGCA). Each assay included a negative control (chicken tissue that has not been injected with human cells,) a positive control (human genomic DNA from SW620 tumour), and the experimental samples in triplicate.

### Fluorescence-activated cell sorting analysis

Cells recovered from CAM tumour tissues after collagenase treatment were washed in DMEM and resuspended in PBS, followed by Percoll purification. GFP-labeled and unlabeled SW620 cells from culture served as controls. Samples were filtered through 35 µm mesh cell strainers (Becton Dickinson) before sorting. GFP-positive cells were collected with a FACSAria machine (Becton Dickinson, San Jose, CA). Data collection was performed with FACSDiva software. On average, between 2 × 10^4^ and 1 × 10^5^ GFP-positive cells were collected. Total RNA of the GFP-positive population was immediately isolated using Trizol LS Reagent (Invitrogen) and subsequently purified by ethanol precipitation. RNA was prepared for microarray analysis using the WT-Ovation Pico RNA amplification system and hybridized to Affymetrix GeneChip Human Gene 1.0 ST arrays using standard protocols (Microarray Core Facility, Center for Functional Genomics, University at Albany). Validation of the Microarray gene expression analysis data was carried out by the quantitative TaqMan PCR assay (Applied Biosystems) using TaqMan Gene Expression Master Mix Reagent Kit according to manufacturers protocols. Following TaqMan Gene Expression primer sets were used: Hs00332747_m1 (*KIAA1456/hTRM9L*), Hs00267016_m1 (*POR*), Hs00167441_m1 (*ALAS1*), Hs00542748_m1 (*LIN9*) or 1× HuGAPDH (Pre-Developed TaqMan Assay Reagents). All data points represent independent biological samples (*N* = 3), with error bars representing standard deviations and statistical significance determined using a Student's *t*-test.

### Colony formation assay

SW620-LacZ and -hTRM9L expressing cells were seeded at a density of 50 cells per well in a 12-well culture dish and cultured for 12 days under either normoxic (21% oxygen) or hypoxic (1% oxygen) conditions. Cells were fixed and stained with crystal violet (Fig 5). All data points represent independent biological samples (*N* = 3) with the average value and standard deviation (error bars) shown in Figures 1F, 2 and 4A.

### Immunohistochemistry (IHC) and immunocytochemistry (ICC)

For anti-phospho histone 3 (pH3), cleaved Caspase-3, LC-3, p21, p27 and GLUT1 IHC, tumours were embedded in an O.T.C. compound, sectioned at 8.0 µm, mounted on Superfrost Plus Slides and fixed in 100% ethanol. The day before immunostaining, the sections were hydrated overnight in a humidified incubator at 37°C. Sections were washed with PBS for 5 min, permeabilized with 0.5% Triton-X in PBS for 10 min, endogenous peroxides were inactivated with 3% hydrogen peroxide for 20 min and blocked with normal goat serum in PBS for 1 h. They were then incubated with the primary antibodies at a dilution of 1:100 overnight at 4°C except for Glut-1 that was used at a dilution of 1–5000. The GLUT1 antibody (Millipore – 07-1401) was a kind gift from Dr. David Burstein (Mount Sinai School of Medicine). Antibodies to Cleaved caspase-3 (Asp175), p-histone H3 (S10), p21^CIP1^, p27^KIP^ were from Cell Signalling Technology and to LC-3 from Axxora, LLC. The biotinylated secondary antibody (Vectastain Elite ABC Kit) was then added and sections incubated for 1 h at RT and detected using a Vectastain ABC Kit following vendor's protocol. Diaminobenzidine solution was then added and sections were incubated for 5 min at room temperature, or until acceptable colour intensity had been reached, after which they were rinsed with deionized water. The nuclei were counterstained with Haematoxilin. For HIF1-α IHC, SW620 LacZ and hTRM9L-expressing cells were grown on cover slips in 35 mm culture dishes (MatTek). Twenty-four hours after exposure to either normoxic or hypoxic conditions, cells were fixed with 4% formaldehyde in PBS for 15 min. Fixed cells were permeabilized and incubated with monoclonal mouse Anti-Human HIF-1α (BD Pharmingen) at a dilution of 1:200 for 2 h. Mouse Anti HIF-1α was detected with Alexa Fluor 488 conjugated goat anti-mouse secondary antibody (Invitrogen) at a dilution of 1:800. Cover slips were mounted with ProLong Gold Antifade Reagent with DAPI nuclear stain (Invitrogen). Fluorescent images were obtained by using a Leica TCS SP5 II confocal inverted microscope with a 63× objective. All data points represent independent biological samples (*N* = 3), with error bars representing standard deviations and statistical significance determined using a Student's *t*-test.

### Cytochemical detection of β-galactosidase staining

Cells from tumour nodules *in vivo* were attached to poly-l-lysine (Sigma)-coated cover slips fixed with 2% formaldehyde, 0.2% glutaraldehyde solution for 15 min. Fixed cells were then stained for the presence of β-galactosidase at pH 6.0 using the Senescence β-galactosidase Staining Kit (Cell Signalling) according to the manufactures protocol. The number of positive stained cells was scored using a Nikon Eclipse TS100 microscope. All data points represent independent biological samples (*N* = 3), with average values shown and error bars representing standard deviations. Statistical significance was determined using a Student's *t*-test.

**The paper explained**

PROBLEM:

The 5-year survival rate for many late stage cancers is very low, as exemplified for stage IV colorectal cancer (∼8%), and new treatment options are desperately needed to stop this disease. Cancer cells in late-stage tumours have turned off important growth inhibitory pathways and deciphering the molecular mechanisms that regulate tumour growth can provide insights for viable therapeutic interventions.

RESULTS:

We have demonstrated that the human tRNA methyltransferase 9-like (hTRM9L) gene is turned off in breast, bladder, testicular, cervical and colon cancers. Modeling this finding in cells derived from late stage colorectal cancers, we demonstrate that reactivation of hTRM9L and its corresponding methyltransferase activity can prevent tumour growth. This occurs via upregulation of LIN9, a known tumour suppressor, and by inhibiting the hypoxic response. Further, we show that hTRM9L deficient colorectal cancer cells are susceptible to killing by aminoglycoside antibiotics and linked this susceptibility to altered tRNA modification levels.

IMPACT:

Aminoglycoside antibiotics are already approved by the Food and Drug Administration to treat gram-positive bacterial infections. Our study suggests that these drugs could be re-purposed to treat some late stage cancers deficient in hTRM9L. Further, hTRM9L levels could serve as a biomarker of susceptibility to these antibiotics, providing a personalized targeted therapy.

### MSD detection of Hif1-α protein levels

Three-day-old tumours were excised from the CAM, minced and resuspended in 0.5 ml of Tris Lysis buffer containing 2× Protease Inhibitor Cocktail (supplied by manufacturer) and then processed on ice using a hand-held tissue homogenizer. Samples were incubated on ice for 15 min before separating the protein extract from cell debris by spinning them for 10 min in a microcentrifuge at 14,000 rpm at 4°C. Protein concentrations were determined by the Bradford assay. A 96-Well MULTI-ARRAY Total HIF1-α Assay was purchased from Meso Scale Discovery (Gaithersburg, MD) and completed according to manufacturer's protocol using 20 µg of total protein. After a 1 h incubation, wells were washed again and 1× MSD Read Buffer was added and plate was analysed immediately by the MSD Sector 2400. All data points represent independent biological samples (*N* = 3). The average value is noted with error bars representing standard deviations.

### Quantification of tRNA modifications

The quantification of RNA modifications in tRNA by liquid-chromatography-coupled mass spectrometry (LC-MS/MS) was performed as described previously (Chan et al, 2010) with several modifications. Following enzymatic hydrolysis and dephosphorylation of purified tRNA, ribonucleosides were resolved on a Thermo Scientific Hypersil GOLD aQ reverse-phase HPLC column (150 × 2.1 mm, 3 µm particle size) eluted with the following gradient of acetonitrile in 8 mM ammonium acetate at a flow rate of 0.3 ml/min and 36°C: 0–18 min, 0%; 18–23 min, 0–1%; 23–28 min, 1–6%; 28–30 min, 6%; 30–40 min, 6–100%; 40–50 min, 100%. The HPLC column was coupled to an Agilent 6410 Triple Quadrupole mass spectrometer with an electrospray ionization source operated in positive ion mode with the following parameters for voltages and source gas: gas temperature, 350°C; gas flow, 10 L/min; nebulizer, 20 psi; and capillary voltage, 3500 V. The first and third quadrupoles (Q1 and Q3) were fixed to unit resolution and the modifications were quantified by pre-determined molecular transitions. Q1 was set to transmit the parent ribonucleoside ions and Q3 was set to monitor the deglycosylated product ions. A dynamic MRM program was set up to monitor modified nucleosides. The retention time, delta time, *m/z* of the transmitted parent ion, *m/z* of the monitored product ion, fragmentor voltage, and collision energy of each modified nucleoside are as follows: D, 2.15 min, 2 min, *m/z* 247 → 115, 80 V, 5 V; acp^3^U, 3.05 min, 2 min, *m/z* 346 → 214, 100 V, 10 V; Y, 3.4 min, 2 min, *m/z* 245 → 125; m^5^C, 4.87 min, 2 min, *m/z* 258→126, 80 V, 8 V; Cm, 5.62 min, 2 min, *m/z* 258 → 112, 80 V, 8 V; m^3^C, 6.33 min, 2 min, *m/z* 258 → 126, 80 V, 8 V; I, 6.44 min, 2 min, *m/z* 269→137, 80 V, 10 V; m^5^U, 6.75 min, 2 min, *m/z* 259 →;127, 80 V, 7 V; ac^4^C, 7.35 min, 2 min, *m/z* 286→154, 100 V, 10 V; ncm^5^U, 7.42 min, 3 min, *m/z* 302 → 170, 90 V, 7 V; m^7^G, 7.91 min, 2 min, *m/z* 298 → 166, 90 V, 10 V; Um, 8.83 min, 2 min, *m/z* 259 → 113, 80V, 7V; m^1^A, 9.33 min, 2 min, *m/z* 282 → 150, 100 V, 16 V; cm5s2U, 9.64 min, 2 min, *m/z* 319187; m1I, 10.17 min, 2 min, *m/z* 283 → 151; mcm^5^U, 11.93min, 3 min, *m/z* 317 → 185, 90 V, 7 V; m^1^G, 14.98 min, 2 min, *m/z* 298 → 166, 90 V, 10 V; m^2^G, 17.23 min, 2 min, *m/z* 298 → 166, 90 V, 10 V; m^5^Um, 19.68 min, 2 min, *m/z* 273 → 127, 100 V, 10 V; Gm, 21.39 min, 3 min, *m/z* 298 → 152; mcm^5^s^2^U, 28.05 min, 3 min, *m/z* 333 → 201, 90 V, 7 V; m22G, yW, 29.5 min, 2 min, m/z 509377; 30.49 min, 2 min, *m/z* 312 → 180, 100 V, 8 V; Am, 32.22 min, 2 min, *m/z* 282 → 136, 100 V, 15 V; OHyW, 36.65 min, 2 min, *m/z* 525 → 393, 100 V, 10 V; and i^6^A, 37.81 min, 2 min, *m/z* 336 → 204, 100 V, 17 V. All data points represent independent biological samples (*N* = 3), with error bars representing standard deviations and statistical significance determined using a Student's *t*-test.

## For more information

Oncomine details for hTRM9L (also called KIAA1456): requires free registration:

https://www.oncomine.org/resource/main.html#dso%3AgeneOverex%3Bec%3A%5B2%5D%3Bepv%3A150001.151078%2C3508%3Bg%3A57604%3Bpg%3A1%3Bpvf%3A7536%5BKIAA1456%5D%3Bscr%3Asummary%3Bv%3A13

RNA Modification Database:

http://rna-mdb.cas.albany.edu/RNAmods/

hTRM9L gene page at NCBI:

http://www.ncbi.nlm.nih.gov/gene/57604

Aguirre-Ghiso lab at MSSM:

http://www.mssm.edu/profiles/julio-a-aguirre-ghiso

Begley Lab at CNSE:

http://cnse.albany.edu/AboutUs/FacultyStaff/Faculty/ThomasBegley.aspx

Dedon lab at MIT:

http://dedon.mit.edu/

## Supplementary material

As a service to our authors and readers, this journal provides supporting information supplied by the authors. Such materials are peer reviewed and may be re-organized for online delivery, but are not copy-edited or typeset. Technical support issues arising from supporting information (other than missing files) should be addressed to the authors.
