# Supplementary material for: A human tRNA methyltransferase 9-like protein prevents tumour growth by regulating LIN9 and HIF1-α
Source: EMBO Mol Med. 2013 Feb 4;5(3):366–83. doi: 10.1002/emmm.201201161 (PMC3598078; doi:10.1002/emmm.201201161)
Supplement: Supplementary file 1 [file emmm0005-0366-SD1.pdf]

EMBO
Molecular Medicine

A human tRNA methyltransferase 9-like protein prevents tumor growth by regulating LIN9 and HIF1α

Ulrike Begley, Maria Soledad Sosa, Alvaro Avivar-Valderas, Ashish Patil, Lauren Endres, Yeriel Estrada, Clement T.Y. Chan, Dan Su, Peter C. Dedon, Julio A. Aguirre-Ghiso and Thomas Begley

Corresponding author: Thomas Begley, University at Albany, State University of New York

Review timeline:

Transfer date:	15 December 2011
Editorial Decision:	25 January 2012
Revision received:	03 May 2012
Editorial Decision:	12 June 2012
Revision received:	16 November 2012
Editorial Decision:	27 November 2012
Revision received:	05 December 2012
Accepted:	07 December 2012

Transaction Report:

(Note: With the exception of the correction of typographical or spelling errors that could be a source of ambiguity, letters and reports are not edited. The original formatting of letters and referee reports may not be reflected in this compilation.)

Editors: Anneke Funk / Roberto Buccione

1st Editorial Decision 25 January 2012

Thank you for the submission of your manuscript "Human tRNA methyltransferase 9 prevents tumor growth by regulating LIN9 and HIF1α" to EMBO Molecular Medicine. We have now heard back from the three referees whom we asked to evaluate your manuscript. You will see that they find the topic of your manuscript potentially interesting. However, they also raise significant concerns on the study, which should be addressed in a major revision of the manuscript.

In particular, it is crucial to rigorously demonstrate that hTRM9 is indeed a tRNA methyltransferase as highlighted by Reviewer #1. In addition, Reviewer #3 points out that more than one cell line should be investigated and that the evidence for induced senescence should be strengthened.

Given the balance of these evaluations, we feel that we can consider a revision of your manuscript if you can convincingly address the issues that have been raised within the space and time constraints outlined below.

Revised manuscripts should be submitted within three months of a request for revision. They will otherwise be treated as new submissions, unless arranged otherwise with the editor.

I look forward to seeing a revised form of your manuscript as soon as possible.

***** Reviewer's comments *****

Referee #1 (Comments on Novelty/Model System):

The authors need to show much more convincingly that hTRM9 is a tRNA methyltransferase.

Referee #1 (Other Remarks):

Referee report on "Human tRNA methyltransferase 9..." by Begley et al

This is a comprehensive study on the role of KIAA1456/hTRM9. First, they show that hTRM9 is downregulated in various cancer types. Interestingly, they show that re-expression of hTRM9 suppress tumor growth in vivo. Of particular interest, they show that hTRM9 expression could modulate sensitivity to paromomycin (and gentamicin) in human cells.

Unfortunately, I am not persuaded that hTRM9 is a tRNA methyltransferase or that hTRM9 catalyzes the last step in the formation of mcm5u. Indeed, the authors show increased level of mcm5u after overexpression of hTRM9 in a cell line expressing low levels of hTRM9 (what about the ABH8 expression in this cell line?). However, these changes are modest, yet significant, and could results from low level contamination of other RNA species or other indirect regulatory mechanisms. There are extremely low levels of the mcm5u precursor, cm5u, in most cells studied. In tumor cells containing less mcm5u one would expect a quite dramatic increase in the amount of cm5u. Was this tested? It is also puzzling that cells lacking ABH8 (Mouse cells) seem to be devoid of the mcm5u, mcm5s2u and mcm5um modifications (in various organs and tRNA isoacceptors;Songe-Moller MCB 2010) - thus, hTRM9 does not seem to be a backup for ABH8. Alternatively, hTRM9 might work on a subset of poorly expressed tRNAs not yet identified to have mcm5u modifications? What about the ribose methylation (mcm5um), could this be the relevant methyl-group formed by hTRM9? There are so many methylation mark in RNA. Although hTRM9 seem to be very similar to yeast TRM9 it might well have other relevant substrates.

Thus, I find that the data indicating that hTRM9 could be a mcm5u methyltransferase is relatively unclear and does not allow for the firm conclusion in the title and throughout the manuscript.

What is TIC (maybe I should have known)? I find no information on this in Material and Methods or in the figure legend (to panel 6B).

MINOR COMMENTS

Introduction; It is stated that "The completion of the wobble uridine modification mcm5U (a precursor for mcm5s2U) is catalyzed by the highly conserved tRNA methyltransferase 9 (Trm9), which has been studied in yeast, mice and humans (Begley et al., 2007; Fu et al., 2010a; Songe-Moller et al., 2010)." It must be indicated here that the mice/human studies relate to ABH8. ABH8 is not TRM9 - but a yeast Trm9 homolog.

It is not correct that ABH8 completes the formation of mcm5u, mcm5s2u and mchm5u and mcm5um (last sentence, first page of introduction). Modify to: "ABH8 make the wobble uridine modifications mcm5U and mchm5U. The formation of mcm5u is required for completion of the mcm5s2U, and mcm5Um modifications."

The authors discuss if the epigenetic silencing of hTRM9 could be directly or indirectly (first page of results). There is a putative CpG island at position: chr8:12611610-12613562 (just at the beginning of the long hTRMP transcript) so this could easily be tested.

Fig 6C
It is somewhat surprising that there is a massive difference in survival already at the lowest concentration of Paromomycin.

Fig 6D
The LacZ expressing cell line (panel D) seems to behave quite differently from the parental SW480 cell line. Would there be a significant difference in survival if comparing SW480 survival shown in panel C with the SW480+hTRM9 survival shown in panel D?

Referee #2:

In this study Begley et al. characterize the role of hTRM9 in tumor suppression. They demonstrated that hTRM9 is lost in various human cancers, and restoration of hTRM9 in colon cancer cells deficiency for hTRM9 inhibited colon cancer development. Mechanistically, they showed that hTRM9 upreguates Lin9 to induce cellular senescence. Moreover, they found that Glut1 expression, a HIF target, is reduced in hTRM9 expressing tumor compared to control tumor. While this study is potentially interest and represents a sufficient advance in the filed, several issues need to be addressed before it can be considered for its publication in EMBO Mol. Med.

Major points:

1. How is hTRM9 lost in human cancers? Since hTRM9 expression is restored by aza-deoxycytidine, does the hTRM9 promoter region get heavily methylated?
2. Since hTRM9 displays t-TRNA methytransferase activity, is its enzymatic activity required for its function in cellular senescence and tumor suppression? To address this question, the enzyme dead mutant should be included in the study.
3. The author demonstrated that Lin9 gene expression is upregulated in hTRM9 expressing cancer cells. Can the authors also show its protein expression should to confirm the Real time PCR result.
4. siRNA usually has off-target effect. To validate the role of Lin9 in hTRM9, at least two sets of siRNAs for Lin9 should be used in Fig. 4D.
5. The authors concluded that HIF response is abrogated in hTRM9 expressing tumor by examining Glut1 expression. Can the authors also show that another HIF target gene is also down hTRM9 expressing tumor.
6. How does hTRM9 regulates hypoxia response? The authors propose a model that LIN9 may be involved in this process. Can the author test whether Lin9 regulates hypoxia response?

Minor Points:

1. The supplementary Figure 1 was not described in the paper.
2. The description in result section for Supplementary Fig. 2-4 are not correct.

Referee #3 (Comments on Novelty/Model System):

Nearly all the worked is performed on a single cell line pair. The authors have not shown much TRM9 they have overexpressed in this SW620 cell line (is it physiological or massively overexpressed?)

Referee #3 (Other Remarks):

In this interesting and potentially important manuscript Begley and colleagues investigate the role of TRM9 as tumour suppressor (principally in colorectal cancer (CRC)).
Overall they present a well thought out series of experiment that show downregulation of TRM9 may contribute to CRC progression. Although i like this manuscript a lot, I have one major concern that they only manipulated TRM9 in one cell line and this needs to be addressed in a revision.

Specific
1. The authors should show the levels of TRM9 they reexpress in the SW620 lines and compare to levels in other CRC cell lines and normal intestine.
2. The authors suggest that there is a downregulation in TRM9 during CRC progression. This data is not completely clear from the data in the paper. Do adenomas have high levels of TRM?
3. The authors provide us with xenograft experiments but with no real characterisation of the proliferation qualities of the cells when they are injected in. For example how healthy are the SW620 cells when they are pre-treated with 5 AZAC. This is more important for the TRM9

experiments as the biggest phenotype is a lag of growth at the beginning, if the cells are unhealthy in vitro then one would expect less growth in vivo as a consequence.
4. Although I am convinced with the growth arrest phenotype of the TRM9 reexpression, whether this is senescence or not is unclear to me?. The authors should also stain for Ki67 and MCM2 which gives a indicator of proliferative capacity of these cells.
5. Following up from points 2 and 4, it would appear quite strange that tumours would have a senescent checkpoint so late in their development most evidence of these checkpoints are early and oncogene induced (eg KRAS induced). Thus i prefer the authors models where in hypoxia TRM9 downregulation would lead to increased proliferation.
6. Following on from this does LIN9 downregulation in TRM9 overexpressing cells rescue the hypoxia phenotype.
7. Are HCT116 cells treated with 5-AZA more sensitive to Paromomycin? One would expect they are?

Minor points
1. What is the magnification of Fig 5A
2. Please improve Fig5E resolution

1st Revision - authors' response 03 May 2012

Referee #1 (Comments on Novelty/Model System):

The authors need to show much more convincingly that hTRM9 is a tRNA methyltransferase... Indeed, the authors show increased level of mcm5u after overexpression of hTRM9 in a cell line expressing low levels of hTRM9 (what about the ABH8 expression in this cell line?). However, these changes are modest, yet significant, and could results from low level contamination of other RNA species or other indirect regulatory mechanisms. There are extremely low levels of the mcm5u precursor, cm5u, in most cells studied. In tumour cells containing less mcm5u one would expect a quite dramatic increase in the amount of cm5u. Was this tested? It is also puzzling that cells lacking ABH8 (Mouse cells) seem to be devoid of the mcm5u, mcm5s2u and mcm5um modifications (in various organs and tRNA isoacceptors;Songe-Moller MCB 2010) - thus, hTRM9 does not seem to be a backup for ABH8. Alternatively, hTRM9 might work on a subset of poorly expressed tRNAs not yet identified to have mcm5u modifications? What about the ribose methylation (mcm5um), could this be the relevant methyl-group formed by hTRM9? There are so many methylation mark in RNA. Although hTRM9 seem to be very similar to yeast TRM9 it might well have other relevant substrates. Thus, I find that the data indicating that hTRM9 could be a mcm5u methyltransferase is relatively unclear and does not allow for the firm conclusion in the title and throughout the manuscript.

These are excellent points and we thank this reviewer for identifying this issue and highlighting pertinent areas related to ABH8 and mcm^5-based tRNA modifications. We understand that in our first submission we too strongly associated yeast Trm9 and KIAA1456 (which we originally termed as hTRM9), with regard to mcm^5-based biochemical activity. We agree with the reviewer that hTRM9 could be something other than a mcm^5U methyltransferase and we have modified the manuscript accordingly. We have generated new data that led us to modify the title, text and figures to address this concern. We agree with the reviewer's characterization of ABH8 (we have converted to ALKBH8 to keep this name constant with the field) as the primary mcm^5-based tRNA methyltransferase in mammalian cells and we have addressed specific potential substrates identified in the reviewer's comments. Ultimately, we have demonstrated that KIAA1456 is a human TRM9-like (hence the new name hTRM9L) protein that promotes the global reprogramming of many tRNA modifications, including an increase in mcm^5U after paromomycin treatment in human cells. We have been unable to directly link hTRM9L's catalytic activity to the formation of mcm^5U based modifications from cm^5U. Using new data derived from our yeast *trm9D* rescue system, which provides the cm^5U substrate *in vivo*, we clearly demonstrate that ALKBH8 will promote the formation of mcm^5-based modifications while hTRM9L cannot. Notably, hTRM9L will promote an increase in the levels of 11 other tRNA modifications and a significant decrease in cm^5U substrate, further linking this protein to tRNA modification. Interestingly, both ALKBH8 and hTRM9L promote a decrease in cm^5U modifications, which ALKBH8 directs into mcm^5U-based modifications and hTRM9L directs into yet to be classified modification(s). Thus we have highlighted ALKBH8 as the sole mcm^5-based tRNA

methyltransferase and put fourth hTRM9L as an activity that will influence the levels of many tRNA modifications. The TRM9-like qualities of KIAA1456 arise from sequence similarity to yeast Trm9, its affect on the cm^5U substrate, its ability to partially rescue the paromomycin sensitivity of *trm9D* cells and its ability to influence the levels of many tRNA modifications. We have substantial evidence to support our claim that hTRM9L plays a role in tRNA modification.

We would like to highlight that the main focus of our manuscript is the hTRM9L-dependent cancer growth and paromomycin sensitivity phenotypes. Identification of hTRM9L as an activity that influences the levels of tRNA modifications and associates these activities to cancer growth regulation provides a novel bridge between tRNA modification and cancer biology. We believe that by editing the manuscript we have also addressed the reviewers concerns regarding the specific classification of hTRM9L biochemical activity. Further, we believe our work makes a relevant discovery by detailing the biological significance of hTRM9L silencing as a potential therapeutic benefit to treat these tumours with aminoglycoside-based therapies.

What is TIC (maybe I should have known)? I find no information on this in Material and Methods or in the figure legend (to panel 6B).

We apologize for our oversight and have added Total Ion Count (TIC) to the figure legend.

MINOR COMMENTS

Introduction; It is stated that "The completion of the wobble uridine modification mcm5U (a precursor for mcm5s2U) is catalysed by the highly conserved tRNA methyltransferase 9 (Trm9), which has been studied in yeast, mice and humans (Begley et al., 2007; Fu et al., 2010a; Songe-Moller et al., 2010)." It must be indicated here that the mice/human studies relate to ABH8. ABH8 is not TRM9 - but a yeast Trm9 homolog.

This is a good point. We have indicated that the mice and human studies relate to ABH8.

It is not correct that ABH8 completes the formation of mcm5u, mcm5s2u and mchm5u and mcm5um (last sentence, first page of introduction). Modify to: "ABH8 make the wobble uridine modifications mcm5U and mchm5U. The formation of mcm5u is required for completion of the mcm5s2U, and mcm5Um modifications."

We apologize for this error and modified as indicated.

The authors discuss if the epigenetic silencing of hTRM9 could be directly or indirectly (first page of results). There is a putative CpG island at position: chr8:12611610-12613562 (just at the beginning of the long hTRMP transcript) so this could easily be tested.

We are currently testing the epigenetic silencing of TRM9 by not only looking at CpG island methylation but also histone post-translational modifications that may cause the heterochromatinization of its promoter, as a recent paper (*GENES, CHROMOSOMES & CANCER 40:247–260 (2004))* showed no strong evidence for methylation dependent repression of hTRM9L expression, via its promoter. This will be part of a more detailed separate study on these mechanisms.

Fig 6C. It is somewhat surprising that there is a massive difference in survival already at the lowest concentration of Paromomycin.

We agree with this observation. We have added the following text to the discussion, “there appears to be a low threshold for sensitivity to paromomycin in hTRM9L-deficient cells”.

Fig 6D. The LacZ expressing cell line (panel D) seems to behave quite differently from the parental SW480 cell line. Would there be a significant difference in survival if comparing SW480 survival shown in panel C with the SW480+hTRM9 survival shown in panel D?

We have determined there is little difference between SW480 and SW620 + hTRM9L after paromomycin treatment.

Referee #2:

Major points:

1. How is hTRM9 lost in human cancers? Since hTRM9 expression is restored by aza-deoxycytidine, does the hTRM9 promoter region get heavily methylated?

Previous evidence (*GENES, CHROMOSOMES & CANCER 40:247–260 (2004))* suggests that certain CpG islands are not differentially methylated between SW480 and SW620 cells or in tumour samples. However, other promoter or enhancer regions that where not tested could be subject to hypermethylation or methylation of the promoter of a positive inducer of hTRM9L could be responsible for its silencing. Ongoing studies are testing the role of CpG island methylation and histone post-translational modifications in regulating hTRM9L silencing and their extent in cancer. These studies will be part of a separate manuscript.

2. Since hTRM9 displays t-TRNA methyltransferase activity, is its enzymatic activity required for its function in cellular senescence and tumour suppression? To address this question, the enzyme dead mutant should be included in the study.

This is an important idea to test. Detailed biochemical and structure function studies are the focus of our next manuscript.

3. The author demonstrated that Lin9 gene expression is upregulated in hTRM9 expressing cancer cells. Can the authors also show its protein expression should to confirm the Real time PCR result.

We have observed a 1.8 fold increase in LIN9 protein levels in hTRM9L proficient cells using Western blots (supplemental figure 3). This observed protein increase confirms our qPCR result and we have added this information to the manuscript.

4. siRNA usually has off-target effect. To validate the role of Lin9 in hTRM9, at least two sets of siRNAs for Lin9 should be used in Fig. 4D.

We have been unable to achieve sufficient knockdown of LIN9 using any of the other RNAi constructs that we tested. We note that we have used a scrambled control to account for off target effects, which is a common standard for knockdown experiments.

5. The authors concluded that HIF response is abrogated in hTRM9 expressing tumour by examining Glut1 expression. Can the authors also show that another HIF target gene is also down hTRM9 expressing tumour.

The tumours are significantly hypo-vascular even from gross analysis of lesions. Glut1 is a prototypical HIF1α target as it contains conserved and well characterized binding elements in its promoter and it has been reported as a robust readout for hypoxia in human samples. We believe that this serves as a good marker for a blunted hypoxic response.

6. How does hTRM9 regulates hypoxia response? The authors propose a model that LIN9 may be involved in this process. Can the author test whether Lin9 regulates hypoxia response?

This is an excellent idea and we are exploring the association between LIN9 and hypoxia in our next manuscript. We are currently generating a LIN9 inducible knockdown system in SW620 (+/- hTRM9L) lines for these experiments. We believe that at this stage this is beyond the focus of the current manuscript but agree with the reviewer that there are exciting mechanistic implications.

Minor Points:

1. The supplementary Figure 1 was not described in the paper.

We have included text in the manuscript to address this concern.

2. The description in result section for Supplementary Fig. 2-4 are not correct.

We apologize for our clerical error and have rectified the description in the manuscript.

Referee #3 (Comments on Novelty/Model System):

I have one major concern that they only manipulated TRM9 in one cell line and this needs to be addressed in a revision.

This is an excellent point and we have addressed it with the generation of two new hTRM9 proficient colorectal lines (HCT116 and HT29). As noted above and in the revised manuscript, we have performed phenotypic tests with HCT116 and HT29 cells, expressing hTRM9L or LacZ, to support our SW620-specific findings. Specifically, we demonstrate that HCT116-hTRM9L expressing cells exhibit decreased tumour growth in nude mice, relative to HCT116-lacZ. In addition, both the hTRM9L expressing cells (HCT116 and HT29) are resistant to paromomycin, relative to lacZ expressing cells. We have added new text and figures to the manuscript to describe these new cell models and supporting data. Ultimately we have demonstrated the general phenotype that hTRM9L can provide resistance to aminoglycoside antibiotics and prevent tumour growth in many colorectal cancer backgrounds.

Minor concerns

1. The authors should show the levels of TRM9 they reexpress in the SW620 lines and compare to levels in other CRC cell lines and normal intestine.

This is a complicated comparison and we note that all of this information was originally included in figure 1C and Table 1. As seen in Fig1C SW480 cells that are hTRM9L+ express ~100 times more hTRM9L mRNA than SW620 cells. HT29 cells express ~20 fold more hTRM9L mRNA than SW620. Table I shows that upon re-expression of hTRM9L there is a 39 fold higher mRNA abundance of hTRM9L compared to LacZ cells. Thus, the overexpression is within range of that observed occurring naturally in colorectal cancer cell lines. Abundant data in Oncomine also supports our findings and shows that hTRM9L is decreased compared to normal colonic epithelium, within the range of what we observe in the CRC cell lines. Further our tumour panel analysis of colorectal tumours vs. normal tissue showed 3 samples with a down regulation of hTRM9L ranging from 20-50 fold decrease, suggesting that the overexpression we are achieving is present in human samples and therefore realistic.

2. The authors suggest that there is a downregulation in TRM9 during CRC progression. This data is not completely clear from the data in the paper. Do adenomas have high levels of TRM?

We apologize for the confusion. We can only conclude that there is a significant decrease of hTRM9L expression with stage in colorectal tumours. Thus, in already established tumours there is a down-regulation of hTRM9L as they progress. We cannot conclude that this down-regulation occurs early during progression (*i.e.,* adenomas).

3. The authors provide us with xenograft experiments but with no real characterization of the proliferation qualities of the cells when they are injected in. For example how healthy are the SW620 cells when they are pre-treated with 5 AZAC. This is more important for the TRM9 experiments as the biggest phenotype is a lag of growth at the beginning, if the cells are unhealthy in vitro then one would expect less growth in vivo as a consequence.

This is a good point and we carefully examined the different engineered cell lines expressing LacZ or hTRM9L or treated with AzaC. Using Trypan Blue exclusion to monitor for cell health, we did not observe any significant difference in cellular viability or proliferation when comparing SW620-LacZ and SW620-hTRM9L expressing cell lines. Cells were always seeded at the same concentration and where at the same confluency on the day they were prepared for xenograft injections. We did not observe significant toxicity when we pre-exposed SW620 and HCT116 colon cancer cell lines to 5-azaC for the indicated time and concentration. This information is now included in the new materials and methods sections as well as stated in results.

4. Although I am convinced with the growth arrest phenotype of the TRM9 reexpression, whether this is senescence or not is unclear to me?. The authors should also stain for Ki67 and MCM2 which gives a indicator of proliferative capacity of these cells.

We have included new text to address this concern. The problem with Ki67 is that it stains cells in all phases of cell cycle except G0. Thus, slow cycling or G0/G1 boundary arrested cells would be scored as proliferative when they are not. That is why we used phospho-H3 that is active only in cells that are going through G2->M. The absence of mitotic figures in hTRM9L expressing cells and the enhanced staining for p21 suggest that this is not a G2/M arrest, but rather a G0/G1 arrest. In our original version we indicated that this was a senescence-"associated" arrest. We never concluded that it is a canonical senescence phenotype. To better define this phenotype, we have used immunohistochemistry to analyse the senescence markers heterochromatin protein 1γ (HP1γ) and

Histone-3-tri-methyl-Lysine-9 in hTRM9L proficient and deficient cells. We noted little or no difference in the levels of these two markers in hTRM9L-proficient and deficient cells and together with our SA-b-gal data we have used this information to describe a senescence-like program. Thus, we conclude that it is a G0-G1 arrest that only partially overlaps with a senescence program. The fact that tumours eventually grow and still express hTRM9L suggests that the arrest is not terminal and that it can be bypassed. Thus, perhaps an overlap between quiescence (reversible arrest) and senescence programs are induced by TRM9. Further experiments will better define these mechanisms

5. Following up from points 2 and 4, it would appear quite strange that tumours would have a senescent checkpoint so late in their development most evidence of these checkpoints are early and oncogene induced (e.g. KRAS induced). Thus I prefer the authors models where in hypoxia TRM9 downregulation would lead to increased proliferation.

This is a good point and we agree with the reviewer's line of reasoning. As described in point 4 we are not convinced that the phenotype is a canonical senescence program. That is why we called it a senescence-associated arrest. We have now used new markers and we have restated the phenotype to read "senescence-like." We have edited to senescence-like arrest in figure 7.

6. Following on from this does LIN9 downregulation in TRM9 overexpressing cells rescue the hypoxia phenotype.

This is an excellent idea and we are exploring the association between LIN9 and hypoxia in our next manuscript. Since the LIN9 RNAi restores tumour growth in hTRM9L-expressing cells and this is dependent on angiogenesis we assume that the reviewer's interpretation is correct. However, we have not fully explored all angles. We are currently generating a LIN9 inducible knockdown system in SW620 lines for these experiments. We believe that this is outside the focus of the current manuscript but agree with the reviewer that there are exciting mechanistic implications.

7. Are HCT116 cells treated with 5-AZA more sensitive to Paromomycin? One would expect they are?

We are unsure if the reviewer's conclusion is correct, as 5-AZA treatment leads to the expression of hTRM9L (Fig. 1D), which should provide resistance to paromomycin. Unfortunately 5-AZA treatment affects many genes, so the best evidence is to directly express hTRM9L back in HCT116. In support of this hypothesis, we have included data specific to HCT116 cells expressing hTRM9L, and relative to HCT116, they are more resistant to paromomycin (Supplementary Figure S5).

Minor points

1. What is the magnification of Fig 5A

10X

2. Please improve Fig5E resolution.

We have increased the size of these images and made sure that these images are in the accepted resolution for the journal.

2nd Editorial Decision 12 June 2012

Thank you for the submission of your revised manuscript "A human tRNA methyltransferase 9-like protein prevents tumor growth by regulating LIN9 and HIF1α" to EMBO Molecular Medicine. We have now received the enclosed reports from the referees that were asked to re-assess it.

As you will see, the reviewers acknowledge that the manuscript was improved during revision. However, while reviewer #3 indicates that that the manuscript is suitable for publication, reviewer #2 still raises technical issues that should be convincingly addressed. While we agree that the use of an additional LIN-9-targeted siRNA would be ideal, we also acknowledge the use of the scrambled control siRNA. As such, we would not consider the additional siRNA compulsory for the acceptance of the manuscript. However, reviewer #2 still raises the concern regarding the contribution of hTRM9 enzymatic activity to the investigated phenotypes, which should be

convincingly addressed. Since we do acknowledge the potential interest of your findings, we would therefore be open to allow a second revision of the manuscript that would address the outstanding issues.
Revised manuscripts should be submitted within three months of a request for revision; they will otherwise be treated as new submissions, unless arranged otherwise with the editor.

I look forward to seeing a revised form of your manuscript as soon as possible.

***** Reviewer's comments *****

Referee #2:

The authors have addressed some of my previous concerns. However, but there are two key points remained to be answered (Point 2 and Point 4). For points 2, the enzymatic activity of hTRM9 on cellular senescence and/or tumor suppression should be examined. For point 4, the authors can not rule out the off-target effect if only one siRNA is used. The authors should do the rescue experiment to prove their notion.

Referee #3 (Comments on Novelty/Model System):

This is an interesting and important area. The authors have added in the extra controls required so this means the model system is now adaquate and the technical quality high.

Referee #3 (Other Remarks):

In this revision Begley and colleagues show silencing of TRM9 drives tumour progression in colorectal cancer. This is an exciting area and the data appears robust.
The authors have answered my major comments and now provide extra CRC cell lines to show that this is a general phenotype. They also answer my more minor comments: health of the xenografted cells, toned down their discussion of senescence etc. Some of the other reviewers comments were not answered.

Overall I think this manuscript is robust and worthy of publication. It would also be suitable as a short report.

2nd Revision - authors' response 16 November 2012

From the Editor

As you will see, the reviewers acknowledge that the manuscript was improved during revision. However, while reviewer #3 indicates that that the manuscript is suitable for publication, reviewer #2 still raises technical issues that should be convincingly addressed. While we agree that the use of an additional LIN-9-targeted siRNA would be ideal, we also acknowledge the use of the scrambled control siRNA. As such, we would not consider the additional siRNA compulsory for the acceptance of the manuscript. However, reviewer #2 still raises the concern regarding the contribution of hTRM9 enzymatic activity to the investigated phenotypes, which should be convincingly addressed. Since we do acknowledge the potential interest of your findings, we would therefore be open to allow a second revision of the manuscript that would address the outstanding issues.

This is a good point and we have generated new data to address. We have used site directed mutagenesis and xenograft experiments to support our conclusion that hTRM9L methyltransferase activity is required to prevent tumor growth. In our new figure 2F and supplemental figure 1C we describe our approach and results in which we individually mutated two amino acids found in hTRM9L's evolutionarily conserved methyltransferase domain. This domain defines methyltransferase enzymes and is required to bind the enzymatic co-factor S-adenosylmethionine (SAM) (Kahlor and Clarke, 2003; Katz et al., 2003). Due to their interaction with SAM, most methyltransferase enzymes share this conserved domain structure consisting of three motifs (I-III)

that bind and position SAM near the active site. hTRM9L, ALKBH8 enzymes and yeast Trm9 have all been reported to contain these conserved methyltransferase motifs (Kahlor and Clarke, 2003; Songe-Moller et al, 2010). We took advantage of this known domain structure and made D91R and I108N mutants in hTRM9L. We note that the amino acids we targeted for mutation are strictly conserved in all characterized mcm^5U methyltransferases **(Supplemental Figure 1C)**. Corresponding xenograft experiments using these hTRM9L mutants (D91R and I108N) demonstrated that the native amino acids are required for tumor growth suppression by hTRM9L **(Figure 2F)**, thus identifying this methyltransferase domain and SAM-binding as a key driver of tumor growth suppression. We have also demonstrated that mutation of a residue outside the methyltransferase defining domain has no effect on the tumor growth phenotype, which is in addition to the SW620-LacZ and SW620-hTRM9L control data for these triplicate and statistically supported xenograft results. We believe that these structure function xenograft studies provide strong additional support for our conclusion that the hTRM9L methyltransferase activity, through SAM-binding and catalysis, is required for tumor growth suppression.

Referee #2:

The authors have addressed some of my previous concerns. However, but there are two key points remained to be answered (Point 2 and Point 4). For points 2, the enzymatic activity of hTRM9 on cellular senescence and/or tumor suppression should be examined. For point 4, the authors can not rule out the off-target effect if only one siRNA is used. The authors should do the rescue experiment to prove their notion.

See above note to the editor detailing added support for the hTRM9L methyltransferase activity being essential for tumor growth suppression.

3rd Editorial Decision 27 November 2012

Thank you for the submission of your revised manuscript to EMBO Molecular Medicine. We have now received the enclosed reports from the Reviewer who was asked to re-assess it. As you will see s/he is now globally supportive and I am pleased to inform you that we will be able to accept your manuscript pending the following final amendments:

Please re-write your "The paper explained" summary for this article, so that it is accessible to non-specialists and specialists alike, by highlighting the medical issue you are addressing (heading: PROBLEM), the results obtained (heading: RESULTS), and their clinical impact (heading: IMPACT). Please refer to any of our published primary research articles for an example. This may be further edited by us to ensure that readers understand the significance and context of the research.

In addition, please correct the following figures:
Fig1, panel B: The "E" on the word "Endomethrium" is partially cut
Fig4 panel B: there is a sign after (SW620-LacZ)

In general, please carefully read and follow the instructions listed below for submission of the final version to ensure rapid acceptance.

Please submit your revised manuscript as soon as possible and in any case no later than two weeks from now.

I look forward to reading a new revised version of your manuscript as soon as possible.

***** Reviewer's comments *****

Referee #2:

The authors have addressed all my concerns. The manuscript is now suitable for publication at EMBO Molecular Medicine.

3rd Revision - authors' response 05 December 2012

From the Editor

1. Please re-write your "The paper explained" summary for this article, so that it is accessible to non-specialists and specialists alike, by highlighting the medical issue you are addressing (heading: PROBLEM), the results obtained (heading: RESULTS), and their clinical impact (heading: IMPACT). Please refer to any of our published primary research articles for an example. This may be further edited by us to ensure that readers understand the significance and context of the research.

We have re-written “The paper explained” summary as directed.

2. In addition, please correct the following figures:

Fig1, panel B: The "E" on the word "Endomethrium" is partially cut. Fig4 panel B: there is a sign after (SW620-LacZ)

We have made these two requested corrections.

3. In general, please carefully read and follow the instructions listed below for submission of the final version to ensure rapid acceptance.

We have carefully read the instructions indicated or uploaded the requested files.
