# Supplementary material for: A human tRNA methyltransferase 9-like protein prevents tumour growth by regulating LIN9 and HIF1-α
Source: EMBO Mol Med. 2013 Feb 4;5(3):366–83. doi: 10.1002/emmm.201201161 (PMC3598078; doi:10.1002/emmm.201201161)

Supplementary Material Table of Contents

Supplementary Table I

Supplementary Table II

Supplementary Table III

Supplementary Table IV

Supplementary Table V

Supplementary Figure S1

Supplementary Figure S2

Supplementary Figure S3

Supplementary Figure S4

Supplementary Figure S5

Supplementary Figure S6

Supplemental Table I. Verification of LIN9 knockdown by qPCR.

		Average	Average			Normalized	
	**shRNA**	**C_q GAPDH**	**C_q hLin9**	**ΔC_q hLin9 - GAPDH**	**ΔC_q Expression**	**$\Delta\Delta$ C_q**	**%KD**
					$2^{\wedge -\Delta Cq}$		$(1-\Delta\Delta C_q)$x100
**SW620 LacZ**	non-targeting control	33.680	33.865	0.185	0.880	1.000	
	shRNA against Lin9	33.634	40.000	6.366	0.012	0.013	**98%**
**SW620 hTRM9L**	non-targeting control	33.763	35.741	1.978	0.254	1.000	
	shRNA against Lin9	33.999	40.000	6.001	0.016	0.061	**93%**

Supplemental Table II. tRNA modification levels and standard deviations in yeast cells expressing yTrm9, hALKBH8 and hTRM9L.

Compound Name	WT (average)	trm9D (average)	trm9D + yTRM9 (average)	trm9D + hTRM9L (average)	trm9D + hALKBH8 (average)	WT (stdev)	trm9D (stdev)	trm9D + yTRM9 (stdev)	trm9D + hTRM9L (stdev)	trm9D + hALKBH8 (stdev)
**mcm5Um**	0.0	0.0	0.0	0.0	0.0	0.0	0.0	0.0	0.0	0.0
**cm5s2U**	0.0	0.2	0.0	0.3	0.2	0.0	0.0	0.0	0.0	0.0
**ncm5Um**	4.5	4.3	4.4	3.4	7.7	0.3	0.3	0.5	0.5	0.7
**cm5U**	0.0	1.3	0.0	0.8	0.8	0.0	0.1	0.0	0.1	0.0
**ncm5U**	12.2	15.6	11.0	15.6	18.8	1.1	1.6	1.0	1.4	0.1
**m?G**	1,382.8	1,718.6	1,957.8	2,681.8	3,214.3	114.5	127.4	220.5	143.4	42.4
**ac4C**	110.7	105.9	97.8	113.9	117.6	5.1	6.8	3.6	4.6	3.1
**m1I**	10.4	8.3	7.7	11.2	11.3	2.8	1.3	0.9	1.0	0.5
**m1A**	2,212.1	2,724.8	2,966.9	4,949.3	5,844.7	121.7	220.9	334.9	246.8	68.9
**m5Um**	0.0	0.0	0.0	0.0	0.0	0.0	0.0	0.0	0.0	0.0
**I**	2,232.9	2,114.6	2,024.5	2,538.7	2,665.2	79.0	147.1	88.4	39.7	17.6
**Um**	19.5	21.5	25.0	24.5	28.9	0.7	0.8	1.2	2.2	1.8
**Cm**	472.3	552.7	628.7	950.3	919.3	21.6	30.1	67.5	20.5	16.9
**m5U**	202.5	212.9	211.4	256.7	308.2	18.9	12.7	19.6	13.3	5.1
**m3C**	368.0	435.1	439.6	591.7	657.6	28.7	28.1	31.5	46.0	10.3
**m5C**	1,016.9	1,025.8	1,037.2	1,644.9	1,815.8	89.9	40.2	103.5	60.6	10.2
**D**	45.5	46.7	48.6	58.4	68.6	7.8	8.7	2.3	1.7	2.5
**Y**	18.1	19.1	22.9	28.2	33.0	2.3	4.4	1.5	0.6	1.1
**yW**	22.8	16.7	13.8	24.0	18.5	4.4	0.8	1.1	2.5	0.7
**t6A**	100.8	83.7	75.9	88.1	98.2	21.3	5.2	2.1	12.7	11.5
**i6A**	90.1	66.0	51.2	46.1	76.4	14.4	5.6	8.9	6.0	9.2
**mcm5s2U**	27.1	0.0	20.8	0.0	3.9	4.0	0.0	2.4	0.0	0.5
**mcm5U**	18.2	0.0	16.1	0.0	5.1	1.6	0.0	1.6	0.0	0.4
**m22G**	1,798.0	1,699.9	1,721.8	2,957.3	4,193.7	208.2	82.9	205.3	412.7	478.9
**m7G**	1,854.1	2,040.8	2,610.9	4,340.1	5,620.3	170.9	162.9	416.8	326.5	182.9
**m2G**	1,077.0	997.6	1,016.4	1,708.9	2,426.1	111.8	48.9	131.8	253.8	130.8
**Gm**	827.6	837.7	1,008.1	1,334.2	1,623.8	14.7	27.3	92.7	121.4	84.9
**Am**	1,148.8	1,381.9	1,751.0	2,276.9	2,265.3	62.7	82.8	106.3	57.6	17.1

Supplemental Table III. tRNA modification log_2-fold changes and significance in yeast cells expressing yTrm9, hALKBH8 and hTRM9L.

Compund Name	log2-fold change (WT / trm9D)	log2-fold change (trm9D + yTRM9 / trm9D)	log2-fold change (trm9D + hTRM9L / trm9D)	log2-fold change (trm9D + hALKBH8 / trm9D)	P-value (WT to trm9D)	P-value (trm9D + yTRM9 to trm9D)	P-value (trm9D + hTRM9L to trm9D)	P-value (trm9D + hALKBH8 to trm9D)
**mcm5Um**	ND	ND	ND	ND	NA	NA	NA	NA
**cm5s2U**	-1.0	-1.0	0.7	0.1	**0.00000**	**0.00000**	0.00840	0.54303
**ncm5Um**	**0.1**	0.0	-0.4	0.8	0.42407	0.76193	0.04838	**0.00137**
**cm5U**	**-1.0**	-1.0	-0.6	-0.7	**0.00002**	**0.00002**	**0.00560**	**0.00147**
**ncm5U**	**-0.4**	-0.5	0.0	0.3	0.03928	0.01354	0.96450	0.02596
**m?G**	**-0.3**	0.2	0.6	0.9	0.02739	0.17912	**0.00096**	**0.00004**
**ac4C**	**0.1**	-0.1	0.1	0.2	0.38245	0.14287	0.16602	0.05364
**m1I**	**0.3**	-0.1	0.4	0.4	0.29355	0.58517	0.03474	0.01930
**m1A**	**-0.3**	0.1	0.9	1.1	0.02442	0.35505	**0.00031**	**0.00002**
**m5Um**	**ND**	ND	ND	ND	NA	NA	NA	NA
**I**	**0.1**	-0.1	0.3	0.3	0.28731	0.41478	**0.00852**	**0.00300**
**Um**	**-0.1**	0.2	0.2	0.4	0.02580	0.01542	0.09769	**0.00300**
**Cm**	**-0.2**	0.2	0.8	0.7	0.01977	0.15003	**0.00005**	**0.00005**
**m5U**	**-0.1**	0.0	0.3	0.5	0.47278	0.91728	0.01451	**0.00027**
**m3C**	**-0.2**	0.0	0.4	0.6	0.04453	0.86158	**0.00732**	**0.00021**
**m5C**	**0.0**	0.0	0.7	0.8	0.88334	0.86718	**0.00012**	**0.00001**
**D**	**0.0**	0.1	0.3	0.6	0.87123	0.72072	0.08278	0.01357
**Y**	**-0.1**	0.3	0.6	0.8	0.73591	0.23029	0.02447	**0.00618**
**yW**	**0.4**	-0.3	0.5	0.1	0.07946	0.02146	**0.00855**	0.04959
**t6A**	**0.3**	-0.1	0.1	0.2	0.24812	0.07360	0.61018	0.11962
**i6A**	**0.4**	-0.4	-0.5	0.2	0.05349	0.07216	0.01395	0.16927
**mcm5s2U**	**1.0**	0.8	0.0	0.1	**0.00029**	**0.00012**	1.00000	**0.00014**
**mcm5U**	1.0	0.9	0.0	0.3	**0.00004**	**0.00006**	1.00000	**0.00002**
**m22G**	0.1	0.0	1.4	1.3	0.49050	0.87221	**0.00663**	**0.00089**
**m7G**	-0.1	0.4	1.1	1.5	0.24261	0.09200	**0.00040**	**0.00001**
**m2G**	0.1	0.0	0.8	1.3	0.32228	0.82813	**0.00887**	**0.00006**
**Gm**	0.0	0.3	0.7	1.0	0.60151	0.03786	**0.00230**	**0.00011**
**Am**	-0.3	0.3	0.7	0.7	0.01774	0.00900	**0.00010**	**0.00005**

ND = not determined
grey shaded boxes represent wild-type normalized numbers, as the denominator was zero

Supplemental Table IV. tRNA modification levels and standard deviations in SW620-hTRM9L vs. SW620-LacZ cells (Untreated and Paromomycin, N = 3).

Compound Name	LacZ (average)	LacZ-Paro (average)	hTRM9L (average)	hTRM9L-Paro (average)	LacZ (stdev)	LacZ-Paro (stdev)	hTRM9L (stdev)	hTRM9L-Paro (stdev)
D	569,970.1	517,344.6	464,189.8	420,747.9	43,933.4	27,683.8	48,940.3	48,114.4
acp3U	34,614.6	33,686.5	33,466.8	33,578.5	2,359.1	1,005.5	1,166.2	2,651.9
m5C	4,549,514.7	4,748,438.6	5,043,406.5	4,983,707.4	32,342.2	63,886.0	334,606.8	144,479.8
Cm	1,042,166.6	1,171,780.4	1,180,874.1	1,431,514.6	17,823.3	156,384.7	66,226.8	280,419.7
m3C	2,341,851.2	2,287,157.2	2,360,371.4	2,434,123.8	110,837.4	54,867.2	38,961.8	204,487.1
I	52,030.4	56,180.5	61,835.6	69,141.5	1,906.9	1,383.1	1,856.0	6,059.2
m5U	197,387.7	187,846.5	189,700.6	194,766.8	7,503.7	6,907.0	2,538.0	16,604.7
ac4C	8,409,953.3	8,507,636.8	8,524,907.8	8,320,567.1	979,260.1	453,647.7	635,100.4	223,564.4
ncm5U	25,230.5	24,487.2	24,970.5	24,449.0	3,428.0	758.1	1,360.5	1,560.7
m7G	11,687,600.7	11,512,586.0	11,996,710.0	12,540,134.5	266,671.4	108,414.4	203,962.4	1,031,722.9
Um	23,354.1	24,219.3	24,817.8	28,556.0	667.0	1,589.8	1,768.1	4,089.7
m1A	25,505,705.9	24,652,977.2	22,417,268.8	20,203,837.8	184,646.2	680,195.6	963,848.5	2,517,777.4
mcm5U	1,596.9	1,375.6	1,520.1	1,832.5	38.1	14.9	81.4	169.3
Gm	774,407.5	932,879.6	1,060,966.8	1,361,785.2	26,289.3	159,815.8	125,765.1	311,387.3
m1G	5,462,700.9	5,396,492.3	5,834,001.2	5,964,107.5	253,297.6	198,883.4	165,837.2	740,883.6
ac4C	189,020.1	201,290.3	219,783.4	258,417.9	12,063.4	4,434.1	14,197.2	11,968.5
m2G	4,243,863.7	4,578,079.6	5,211,335.0	5,677,532.7	225,349.8	43,782.3	163,366.1	685,896.3
m5Um	15,522.0	15,666.8	16,290.7	16,460.3	405.7	267.6	479.0	2,034.2
15N5-dA	101,036.6	112,178.2	105,684.2	104,280.2	5,508.3	5,846.1	10,677.5	6,476.6
m22G	3,976,493.2	4,007,521.6	4,231,332.3	4,364,844.7	217,673.8	137,794.5	53,050.2	451,996.6
t6A	232,029.8	237,444.3	278,137.2	325,941.6	16,688.7	3,870.9	11,416.1	37,972.5
Am	409,079.2	649,943.9	642,903.9	1,212,295.6	85,495.0	446,896.5	256,783.3	930,758.6
OHyW	11,039.7	10,973.3	10,721.3	11,555.1	703.8	328.1	770.5	1,056.3
Am	21,695.8	22,957.7	22,312.1	21,296.0	1,928.8	2,015.1	1,862.8	1,294.8
i6A	53,323.1	51,671.9	56,880.5	57,183.7	3,454.0	5,906.5	2,849.7	4,376.0
mcm5s2U	4,333.1	3,178.4	2,845.2	2,573.2	1,856.0	134.1	266.8	151.2

Supplemental Table V. tRNA modification levels in SW620-hTRM9L vs. SW620-LacZ cells, log2-fold changes and significance (Untreated and Paromomycin, N = 3).

Compound Name	log2-fold change (hTRM9L to LacZ) untreated	log2-fold change (hTRM9L to lacZ) Paro	log2-fold change (LacZ Paro / LacZ)	log2-fold change (hTRM9L Paro / hTRM9L)	P-value (hTRM9L to LacZ) untreated	P-value (hTRM9L to lacZ) Paro	P-value (LacZ Paro / LacZ)	P-value (hTRM9L Paro / hTRM9L)
**D**	-0.3	-0.3	-0.1	-0.1	**0.050**	**0.039**	0.154	0.334
**acp3U**	0.0	0.0	0.0	0.0	0.293	0.951	0.565	0.950
**m5C**	0.1	0.1	0.1	0.0	0.057	0.061	**0.009**	0.791
**Cm**	0.2	0.3	0.2	0.3	**0.029**	0.234	0.227	0.206
**m3C**	0.0	0.1	0.0	0.0	0.420	0.296	0.486	0.573
**I**	0.2	0.3	0.1	0.2	**0.022**	**0.023**	**0.038**	0.117
**m5U**	-0.1	0.1	-0.1	0.0	0.137	0.542	0.180	0.629
**ac4C**	0.0	0.0	0.0	0.0	0.420	0.557	0.883	0.627
**ncm5U**	0.0	0.0	0.0	0.0	0.460	0.971	0.732	0.685
**m7G**	0.0	0.1	0.0	0.1	0.164	0.161	0.352	0.421
**Um**	0.1	0.2	0.1	0.2	0.176	0.162	0.434	0.220
**m1A**	-0.2	-0.3	0.0	-0.1	**0.013**	**0.042**	0.104	0.228
**mcm5U**	-0.1	0.4	-0.2	0.3	0.069	**0.010**	**0.001**	**0.045**
**Gm**	0.5	0.5	0.3	0.4	**0.021**	0.101	0.165	0.196
**m1G**	0.1	0.1	0.0	0.0	0.129	0.269	0.740	0.781
**ac4C**	0.2	0.4	0.1	0.2	**0.022**	**0.001**	0.174	**0.023**
**m2G**	0.3	0.3	0.1	0.1	**0.015**	0.050	0.065	0.316
**m5Um**	0.1	0.1	0.0	0.0	**0.002**	0.540	0.633	0.895
**15N5-dA**	0.1	-0.1	0.2	0.0	0.307	0.192	0.074	0.855
**m22G**	0.1	0.1	0.0	0.0	0.117	0.260	0.845	0.638
**t6A**	0.3	0.5	0.0	0.2	**0.025**	**0.016**	0.613	0.105
**Am**	0.7	0.9	0.7	0.9	0.152	0.399	0.411	0.365
**OHyW**	0.0	0.1	0.0	0.1	**0.050**	0.414	0.889	0.331
**Am**	0.0	-0.1	0.1	-0.1	0.335	0.296	0.477	0.481
**i6A**	0.1	0.1	0.0	0.0	**0.046**	0.264	0.697	0.925
**mcm5s2U**	-0.6	-0.3	-0.4	-0.1	0.130	**0.007**	0.343	0.199

Supplemental Figure Legends

Figure S1. (A) SW620 cells expressing either *LacZ* or *hTRM9L* were inoculated in nude mice and tumors were measured every 2 days, up to 11 days. Day 11 data for all tumors is shown. **(B)** Graphs showing quantitative PCR (qPCR) analysis of hTRM9L mRNA transcript levels normalized to h36B4. RNA was purified from LacZ and Trm9 SW620 tumors grown on BALB/c nude mice (N = 10). Cells suspensions were prepared to a concentration of 375,000 cells in 100μl PBS++ and injected in two different sites per animal, in the neck and the flank. **(C)** CLUSTAL based amino acid alignment of hTRM9L homologs. The sequence conserved domain, representing motifs I-III and post motif I (Kahlor and Clarke, 2003; Katz et al., 2003), which define methyltransferase enzymes and are used to interact with SAM is marked by the diamond tipped line. We note that that there is a break in the sequence (….) with the last panel specific to the C-terminal region of hTRM9L and other homologs.

Figure S2. IHC staining results. For panels A to D tumor nodules of SW620-LacZ and SW620-hTRM9L expressing cells were harvested 2 to 5 days post-inoculation and stained for **(A)** caspase 3, **(B)** LC3, **(C)** phospho H3 **(D)** p21. **E.** Tumor nodules of SW620-GFP (grey bars) and SW620-hTRM9L (black bars) expressing cells were harvested 2 to 5 days post-inoculation and stained for acidic β-gal as in Figure 3B. **(F)** HP1γ and H3K9me3 staining in SW620-GFP and SW620-hTRM9L cells.

Figure S3. CAM metastasis data and LIN9 protein levels. (A) LacZ- and hTRM9-SW620 cells do not differ in their intravasating capacity. GFP-SW620 overexpressing hTRM9 or LacZ (1x106 cells) were inoculated onto CAMs. For details see (Kim et al., 1998) After 48 and 72 hrs, the CAM tissue on the opposite side of the embryo (lower CAM, where the cells can only arrive if they entered the vasculature) was analyzed by fluorescence microscopy to detect GFP-positive cells, which were counted in collagenase suspensions of this tissue, using a conventional hemocytometer. Note that there is no difference in the efficiency of intravasation between LacZ- and hTRM9-SW620 cells **(B)** Colonization of chick embryo livers was not affected by hTRM9 gene in SW620 cells. The livers were collected from the same embryos described in A. After incubation with collagenase, DNA from these liver cell suspensions containing putative disseminated tumor cells were extracted using Extract-N-Amp kit. Then human-specific Alu expression was determined by quantitative PCR. Note that there is no difference in the detection of levels of Alu-sequence in the livers of embryos carrying LacZ- and

hTRM9-SW620 cells on the CAM. **(C)** LIN9 protein levels in SW620-LacZ and SW620-hTRM9L expressing cells were determined by immunoblotting. LIN9 bands were quantitated by densitometry and normalized to GAPDH to demonstrate a 1.8-fold increase in LIN9 in hTRM9L-proficient cells.

Figure S4. Quantitative analysis of GLUT1 levels. Tumor nodules of SW620-LacZ (black bar) and SW620-hTRM9L (white bar) expressing cells were harvested and stained for GLUT1 as in Figure 5E. The percentage of GLUT1 positive cells was determined and a T-test was used to determine significance.

Figure S5. Cell viability of SW620-hTRM9L and SW480 after paromomycin treatment. SW480 (blue diamonds) and SW620-hTRM9L (red squares) data from Figure 6 were re-plotted to compare viability after paromomycin exposure.

Figure S6. Cell viability of SW620, HCT116 and HT29 cell lines after treatment with aminoglycoside antibiotics. (A) SW620-LacZ (blue diamonds) and SW620-hTRM9L (red squares) cells were exposed to gentamicin and the percent viability for each was determined by trypan blue staining 24 hours post exposure. **(B)** HT29-LacZ (blue triangles) and HT29-hTRM9L (red squares) cells were exposed to paromomycin and the percent viability for each was determined by trypan blue staining 24 hours post exposure. **(C)** HCT116-LacZ (blue diamonds) and HCT116-hTRM9L (red squares) cells were exposed to paromomycin and the percent viability for each was determined by trypan blue staining 24 hours post exposure.

Supplemental Figure 1

A

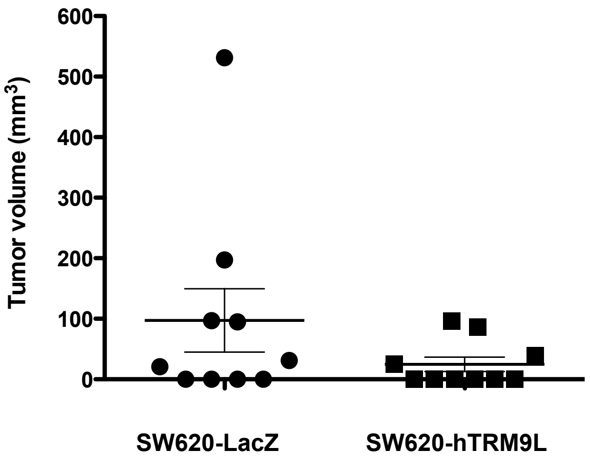

B

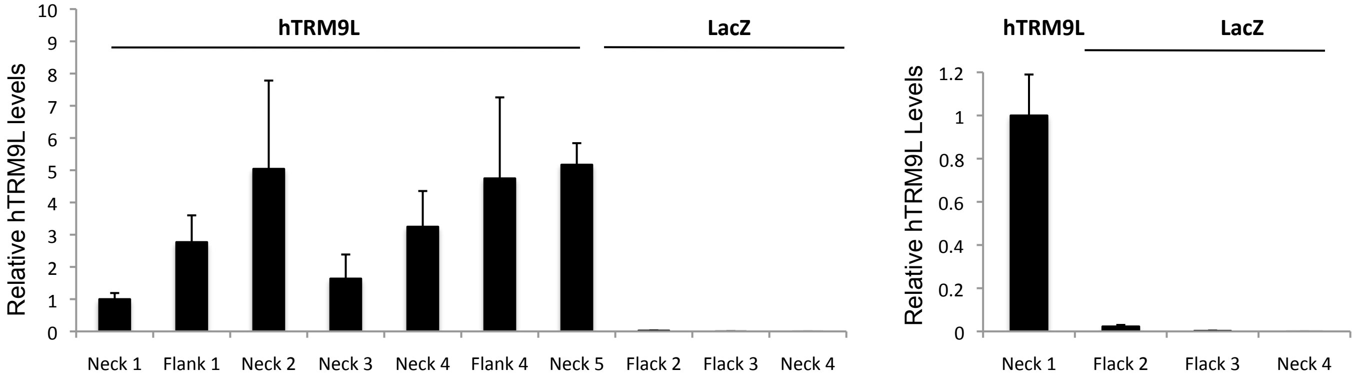

C

```
gi|153251913|ref|NP_065895.2|human TRM9L   (1-454)      1 -MDHEAAQLEKQHVHNVYESTAPYFSDLQSKAWPRVRQFLQEQKPGSLIADIGCGTGKYL
gi|40254307|ref|NP_795926.2| mouse TRM9L   (1-447)      1 -MDPEAVELEKRHVHSVYENTAPYFTDLQSKAWPRVRQFLQDQKPGSLVADIGCGTGKYL
gi|1730616|sp|P49957.1|      yeast TRM9    (1-279)      1 MEINQAAEKEQEYVHKVYNEIAPHFSQTRYKPWPIVTQFLKTRPMGSIGIDVGCGNGKYL
gi|189027650|sp|Q96BT7.2|    human ALKBH8  (362-664) 362 ESDKEASRLEQEYVHQVYEEIAGHFSSTRHTPWPHIVEFLKALPSGSIVADIGCGNGKYL

gi|153251913|ref|NP_065895.2|human TRM9L   (1-454)     60 KVNSQVHTVGCDYCGPLVEIARN--RGCEAMVCDNLNLPFRDEGFDAIISIGVIHHFSTK
gi|40254307|ref|NP_795926.2| mouse TRM9L   (1-447)     60 KVNSQVHTLGCDYCGPLVEIARN--RGCEVMVCDNLNLPFRDQGFDAIISIGVIHHFSTK
gi|1730616|sp|P49957.1|      yeast TRM9    (1-279)     61 GVNPDIYIIGSDRSDGLIECARGINPSYNLLVADGLNLPHKNETFDFAISIAVVHHWSTR
gi|189027650|sp|Q96BT7.2|    human ALKBH8  (362-664) 422 GINKELYMIGCDRSQNLVDICRE--RQFQAFVCDALAVPVRSGSCDACISIAVIHHFATA
                                                                                      * D91R              * I108N

gi|153251913|ref|NP_065895.2|human TRM9L   (1-454)    118 QRRIRAIKEMARVLVPGGQLMIYVWAMEQKNRHF-----EKQDVLVPWNRALCSQLFSES
gi|40254307|ref|NP_795926.2| mouse TRM9L   (1-447)    118 ERRIRAIKEMARVLAPGGQLMIYVWAMEQKNRRF-----EKQDVLVPWNRALCSRLLSES
gi|1730616|sp|P49957.1|      yeast TRM9    (1-279)    121 ERRVEVIRHVLSKLRQGGQALIYCWALEQGSSRRGYHEGMEQDVFVPWVLPKSKSK----
gi|189027650|sp|Q96BT7.2|    human ALKBH8  (362-664) 480 ERRVAALQEIVRLLRPGGKALIYVWAMEQEYNKQ-----KSKYL-------------RGN

.......

gi|153251913|ref|NP_065895.2|human TRM9L   (1-454)    352 STNTGVNCVDAGNIEDDNPSASKILRRISAVDSTDFNPDDTMSVEDPQTDVLDSTAFMRY
gi|40254307|ref|NP_795926.2| mouse TRM9L   (1-447)    349 ITDTGDSVAA---SNSSDPSARKILRRVSAFDSNDSNSEDSSF-LEAQRDATDSKAFMRY
gi|1730616|sp|P49957.1|      yeast TRM9    (1-279)    221 --------------------------------DDND--------EKQQQDQEQEREEVKYRY
gi|189027650|sp|Q96BT7.2|    human ALKBH8  (362-664) 601 -----------------NPDKGKPV-----------------EPFGPIGSQDPSPVFHRY
                                                                                                   R410E *
gi|153251913|ref|NP_065895.2|human TRM9L   (1-454)    412 YHVFREGELCSLLKENVSELRILSSGNDHGNWCIIAEKKRGCD
gi|40254307|ref|NP_795926.2| mouse TRM9L   (1-447)    405 YHVFREGELSSLLQESVSELQVLSSGNDHGNWCIIAEKKRSWD
gi|1730616|sp|P49957.1|      yeast TRM9    (1-279)    243 YHLYREGELAEDCRQAG--AAVHSEGFERDNWWVVAQKR----
gi|189027650|sp|Q96BT7.2|    human ALKBH8  (362-664) 627 YHVFREGELEGAC-RTVSDVRILQSYYDQGNWCVILQKA----
```

Supplemental Figure 2

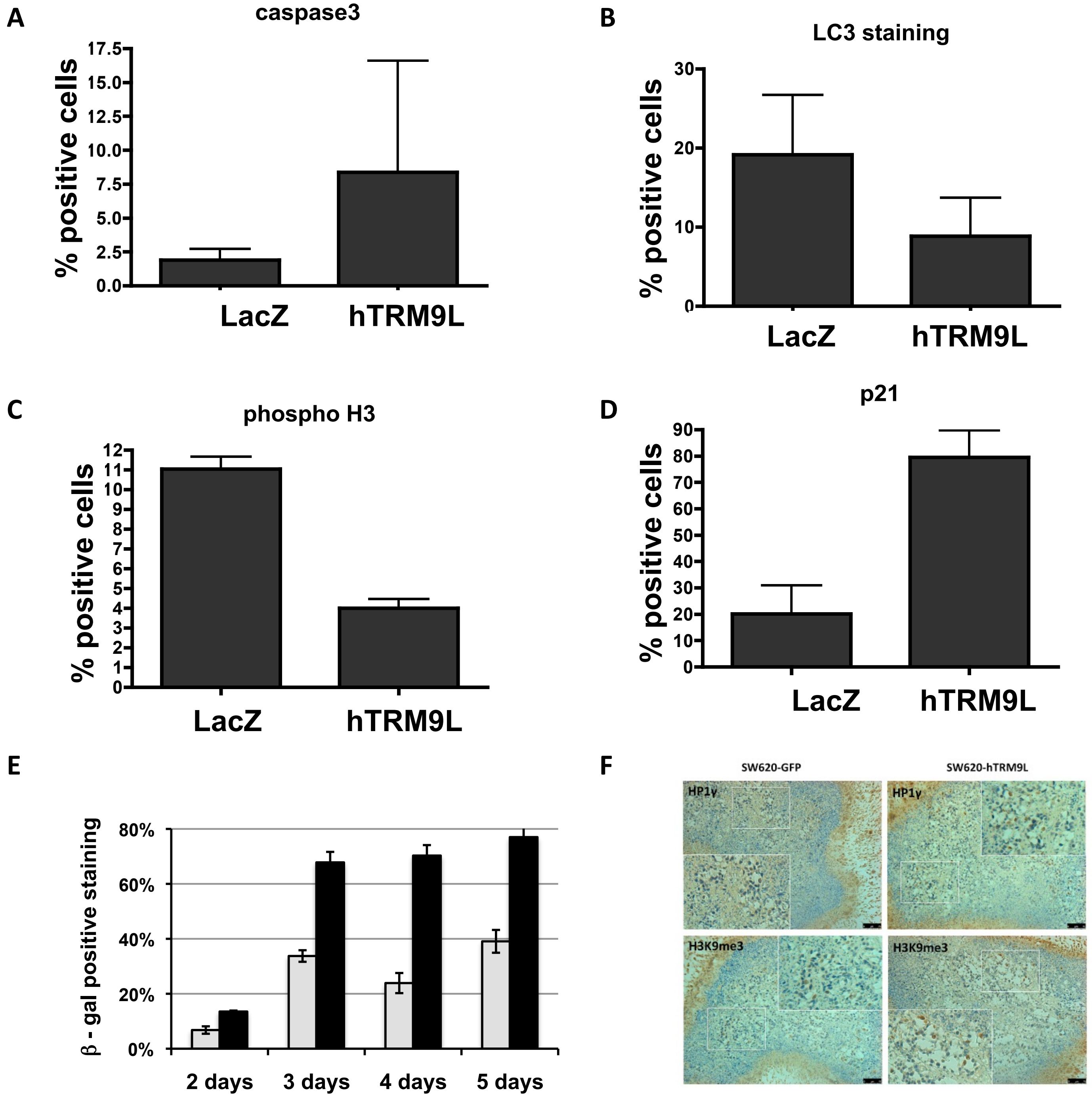

Supplemental Figure 3

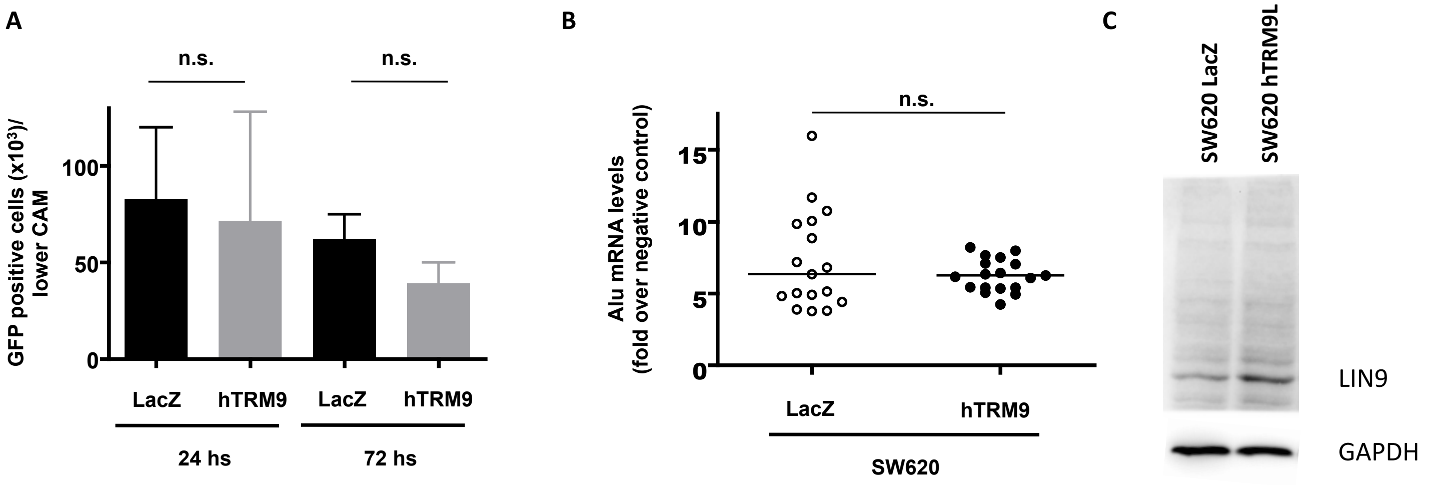

Supplemental Figure 4

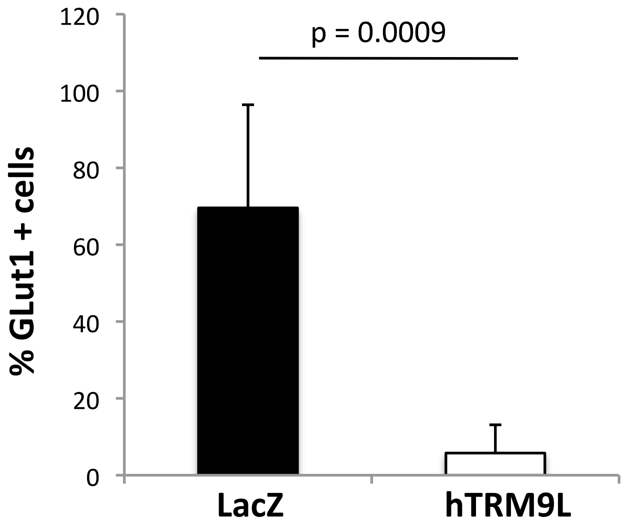

Supplemental Figure 5

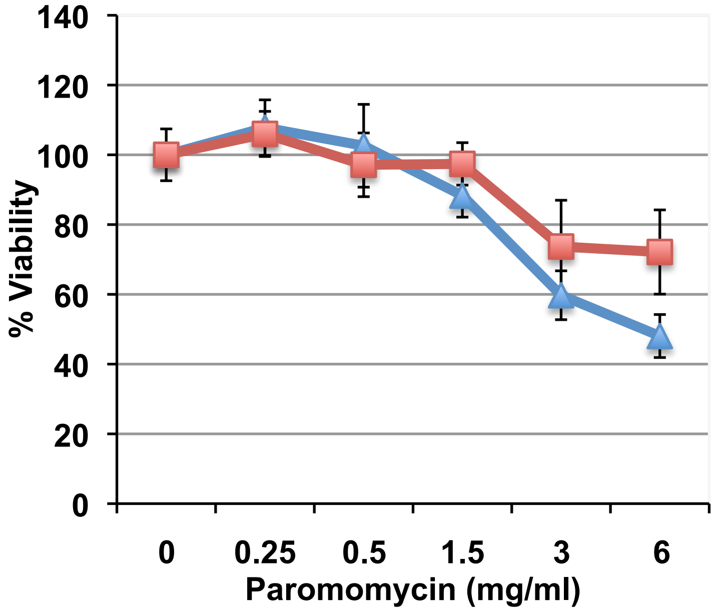

Supplemental Figure 6

A

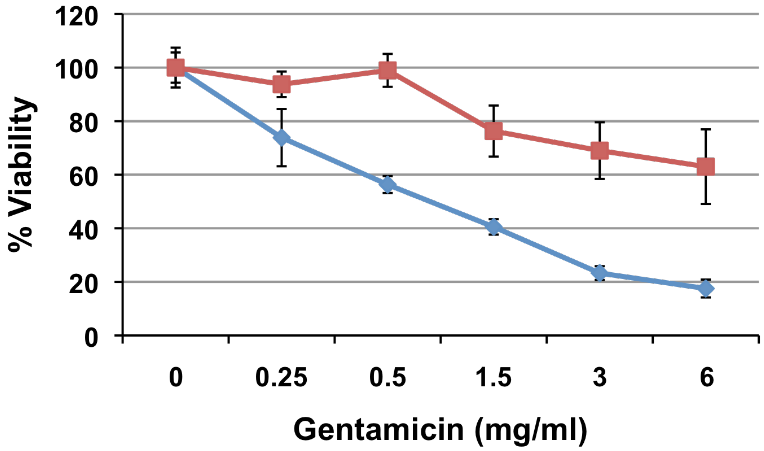

B

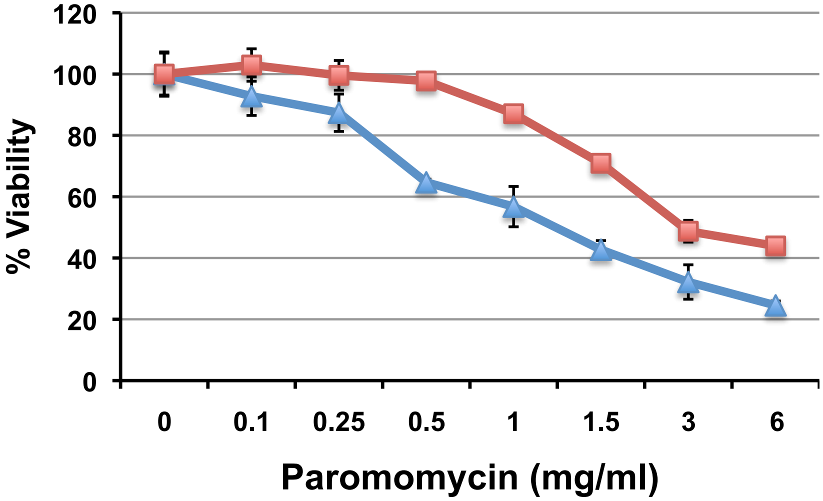

C

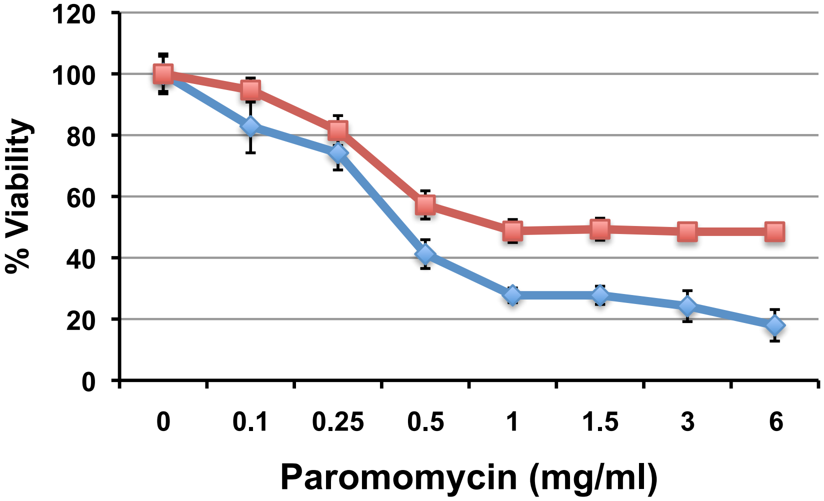

Supplement: Supplementary file 2 [file emmm0005-0366-SD2.pdf]
